# Comparative efficacy of topical commercial Chinese polyherbal preparation for vulvovaginal candidiasis: a network meta-analysis

**DOI:** 10.3389/fphar.2025.1484325

**Published:** 2025-02-03

**Authors:** Lizheng Wu, Shangwen Jing, Na Li, Dandan Cao, Fangli Pei, Yantong Luo, Xiaoxin Chen, Yingjie Huang, Cheng Zeng

**Affiliations:** ^1^ The First Clinical Medical College, Guangzhou University of Chinese Medicine, Guangzhou, Guangdong, China; ^2^ Science and Technology Innovation Center, Guangzhou University of Chinese Medicine, Guangzhou, Guangdong, China; ^3^ The Basic Medicine College, Guangzhou University of Chinese Medicine, Guangzhou, Guangdong, China; ^4^ Affiliated Hospital of Jiangxi University of Traditional Chinese Medicine, Nanchang, Jiangxi, China; ^5^ Department of Gynecology, The First Affiliated Hospital of Guangzhou University of Chinese Medicine, Guangzhou, Guangdong, China

**Keywords:** vulvovaginal candidiasis, commercial Chinese polyherbal preparation (CCPP), Chinese patent medicine, traditional Chinese medicine (TCM), anti-fungal drugs, network meta-analysis, a review

## Abstract

**Objective:**

To systematically evaluate the differences in the efficacy of commonly used topical commercial Chinese polyherbal preparations (CCPPs) for vulvovaginal candidiasis (VVC), and to provide evidence-based reference for clinical drug regimens.

**Methods:**

Computer searched major Chinese and English literature databases, and collected randomized controlled trials (RCTs) of external use of CCPPs combined with conventional treatment (CT, test group) versus CT alone (control group) in patients with VVC. After screening the literature, the quality of the included studies was assessed using the risk of bias assessment tool recommended in 5.3 of the Cochrane Handbook. Outcome data for each outcome measure were extracted and a network meta-analysis was performed using Stata 15.0.

**Results:**

A total of 74 RCTs with a total of 8,151 patients were included, nine interventions were involved. The results of network meta-analysis showed that, Fufang Shajiziyou suppository combined with CT was more effective in improving the negative conversion rate and controlling the recurrence rate of *Candida* albicans. Kangfu gel combined with CT had the better effect in relieving pruritus. Baofukang suppository combined with CT was the most effective in reducing leukorrhea. Fufukang spray combined with CT had fewer adverse reactions.

**Conclusion:**

The addition of CCPPs to CT may acquire a better curative effect in patients with VVC and external does not significantly increase the occurrence of adverse reactions. However, due to the low quality of the included literature, larger-scale, higher-quality clinical studies are still needed.

**Systematic Review Registration:**

PROSPERO, NO: CRD42023410244.

## 1 Introduction

Vulvovaginal candidiasis (VVC) is a prevalent inflammatory gynecologic dermatomycosis resulting from an overgrowth of *Candida* albicans (C. albicans), characterized by a high occurrence and recurrence rate (Saxon Lead Author et al., 2020). Studies indicate that 75% of women will experience at least one episode of VVC in their lifetime, and 40%–50% experiencing multiple episodes (Yano et al., 2019; Sun et al., 2023). Clinical manifestations of VVC include abnormal itching of the vulva and vagina, increased thick leukorrhea resembling tofu curds, and superficial pain during sexual intercourse and so on, all of which significantly impact the quality of life and reproductive health of patients. Current treatment primarily involves oral or intra-vaginal anti-fungal drugs, or through nebulization with ozone insufflation, etc (Denison et al., 2020; Sanjaya et al., 2023). However, these treatments may lead to recurrence of symptoms after cessation and potential side effects such as drug resistance, liver and kidney damage, respiratory irritation, memory loss, or other allergic reactions. Therefore, the need for improved treatment options remains (Satora et al., 2023). Therefore, exploring a better treatment is still the focus of clinical research.

VVC belongs to the category of “vulval pruritus” or “leukorrhagia” based on the theoretical principles of traditional Chinese medicine (TCM), which believes that the core pathogenesis of VVC is dampness-heat in lower jiao, and the therapeutic principle often involves clearing heat and expelling dampness, killing worms and relieving itching (Yang et al., 2018; Feng et al., 2024), the external application of TCM is one of the important treatment methods. As one of the modern TCM preparations, topical commercial Chinese polyherbal preparations (CCPPs) is more stable, easy to store and use, and has been shown in numerous clinical studies to be effective in reducing itching, improving the negative conversion rate and decreasing the recurrence rate of C. albicans (Lijuan et al., 2022). Basic studies have further confirmed the effectiveness, such as the study found that Baofukang suppository (BFKS) can effectively inhibit the adhesion, mycelial formation, and proliferation of C. albicans, restore the morphology and vitality of vaginal epithelial cells, and enhance local immune function (Li et al., 2016). Nevertheless, CCPPs available on the market is booming and there is a lack of research on their relative effectiveness, which is not conducive to their further popularization and application.

Network meta-analysis (NMA) enables simultaneous comparison of three or more interventions for the same disease, offering valuable insights by combining direct comparisons and indirect comparisons and ranking intervention effectiveness, especially when direct comparative studies are insufficient or lacking (Yang et al., 2023). Widely applied in medical research, NMA helped Zhang et al. find Zhibitai capsule as the most effective choice of CCPPs for non-alcoholic fatty liver disease (Zhang et al., 2022). Similarly, Huang et al. found the combination of Shensong Yangxin capsule with Western medicine is presumably the optimal treatment prescription for stable angina through NMA (Huang et al., 2022). Based on these, the study aimed to compare the effectiveness and safety of commonly used topical CCPPs combined with conventional treatment (CT) in treating VVC through NMA to provide evidence for clinical drug use.

## 2 Methods

The study was registered in the International Systematic Review Prospective Register (PROSPERO, NO: CRD42023410244) and carried out according to the Preferred Reporting Items for Systematic reviews and Meta-Analyses (PRISMA) guidelines for systematic reviews and meta-analyses (Page et al., 2021).

### 2.1 Inclusion criteria

The Population-Intervention-Comparators-Outcomes-Study design (PICOS) framework was used as the criterion for this study.

#### 2.1.1 Selection of studies

Publicly published randomized controlled trials (RCTs) with no restrictions on allocation concealment and blinding were included. Language was restricted to Chinese and English.

#### 2.1.2 Study populations

Patients diagnosed with VVC, characterized by symptoms such as vulvovaginal pruritus and curd-like vaginal secretions, and confirmed through the detection of fungal spores or mycelium in the secretions, were included in the study (Nyirjesy et al., 2022), and There were no restrictions regarding age, occupation, race, time of onset, or duration of disease.

#### 2.1.3 Intervention measures

The patients in both groups (control group and test group) were given CT, such as oral or intra-vaginal anti-fungal drugs (clotrimazole preparations, miconazole preparations, nystatin preparations,etc.), 5% sodium bicarbonate solution (vulvar wash) or lacidophilin vaginal capsule. Based on a comprehensive literature review and practical clinical application, the test group was treated with one of eight common topical CCPP, including BFKS, Fufang Shajiziyou suppository (FFSJZYS), Kushen gel (KSG), Kangfu gel (KFG), Fufukang spray (FFKS), Honghe Fujie lotion (HHFJL), Bai’an lotion (BAL) and Jieeryin lotion (JEYL) in addition to the control group’s treatment. The dose and course of treatment were not limited for both groups. Composition, extract and extraction process and more information of PPCCs are shown (Supplementary Tables S1–S3).

### 2.2 Outcomes

1) The negative conversion rate of C. albicans; 2) the recurrence rate of C. albicans; 3) time to resolution of pruritus; 4) time to resolution of secretion; 5) incidence of adverse reactions.

### 2.3 Exclusion criteria

Articles meeting the following criteria were excluded: non-clinical trials, such as reviews, cell experiments and animal modeling experiments, conference reports, empirical summaries, dissertation, treatment guidelines, etc.; relevant outcome indicators were not provided; the full text could not be obtained; the test group only used CCPPs; the clinical data were incomplete and the request for the data from the original author was fruitless; patients with bacterial or trichomoniasis infection; repeatedly published articles, non-Chinese and English literature.

### 2.4 Search strategy

PubMed, CNKI, Wanfang Data, VIP, CBM, Web of science, Cochrane Library and Embase database were searched by computer (Song et al., 2024). According to the characteristics of different databases, MeSH terms, free words and keywords were used for retrieval, and the language of the literature was limited to Chinese and English. The retrieval time was from the inception of each database to February 2024. The search strategy’s complete details are displayed (Supplementary Tables S4–S11).

### 2.5 Data extraction

When screening the literature, the two researchers independently read the titles and abstracts of the articles for primary selection and deduplication, and then read the full text for deciding whether to include or not according to the inclusion and exclusion criteria strictly (Wu et al., 2022). If there is a difference between the two ideas, disagreements were resolved by discussion first or, if necessary, a third-party opinion was consulted to reduce the selection bias. All data were independently extracted through standard tables, including: 1) basic information of the included studies (the first author and year of publication); 2) basic information of the research subjects (sample size and age); 3) intervention measures; 4) course of treatment; 5) outcome indicators (negative conversion rate, recurrence rate, time to improve itching, time to reducing secretion, and occurrence of adverse effects); 6) quality assessment and methodological information of the articles.

### 2.6 Handling of missing data

If data was missing or incomplete, calculated it if possible; for instance, if the data was presented in graphical form, the data could be extracted using GetData software. If calculation failed, contacted the first or corresponding author. Excluded the data from analysis if it remains unavailable.

### 2.7 Risk of bias assessment

The quality of the included RCT was assessed using the quality assessment methods recommended in the Cochrane Handbook for Systematic Evaluators 5.3 (Higgins et al., 2011), which include the following seven items: 1) generation of the randomized sequence; 2) allocation concealment; 3) blinding of participants and personnel; 4) blinding of outcome measures; 5) completeness of data on outcome indicators; 6) selective reporting; 7) other bias. For the included articles, each item was judged as “Yes” (low risk), “No” (high risk), or “Unclear” (unclear risk). Methodological quality was assessed independently by two reviewers, and in case of disagreement, a third-party opinion was reached.

### 2.8 Statistical analysis

Data were statistically analyzed and graphically presented using Stata15.0 software (Shim et al., 2017), and continuous variables were expressed by standardized mean difference (SMD) and 95% confidence interval (CI), and dichotomous variables were expressed by odds ratio (OR) and 95% CI. Heterogeneity test used I^2^ to test the magnitude of heterogeneity among the studies, if I^2^ ≤ 50%, it was considered that there was no statistical heterogeneity among the included studies, and the fixed-effects model was used for meta-analysis; if I^2^ > 50%, it was suggestive of the existence of heterogeneity among the studies, and the random-effects model was selected for meta-analysis. In outcomes with significant heterogeneity, subgroup analyses were performed to identify potential sources of heterogeneity. Descriptive analyses were used when the source of heterogeneity could not be determined.

If there was a closed loop in a network, the consistency between direct and indirect comparisons was first compared using the node-split model, with *P* > 0.05 indicating good consistency, and the consistency model was employed for analysis, while the inconsistency model was utilized for the converse scenario. In studies where node-split nodes were not produced, the consistency model was first applied for analysis. The cumulative ranking probability of each therapy was determined through the calculation of the surface under the cumulative ranking curve area (SUCRA), with higher SUCRA values indicating superior intervention efficacy. Comparison-corrected funnel plots were drawn with Stata 15.0 to determine whether there was publication bias or small sample effects. Sensitivity analysis was used to evaluate the stability of meta-analysis results.

## 3 Results

### 3.1 Literature retrieval and basic information

**FIGURE 1:**
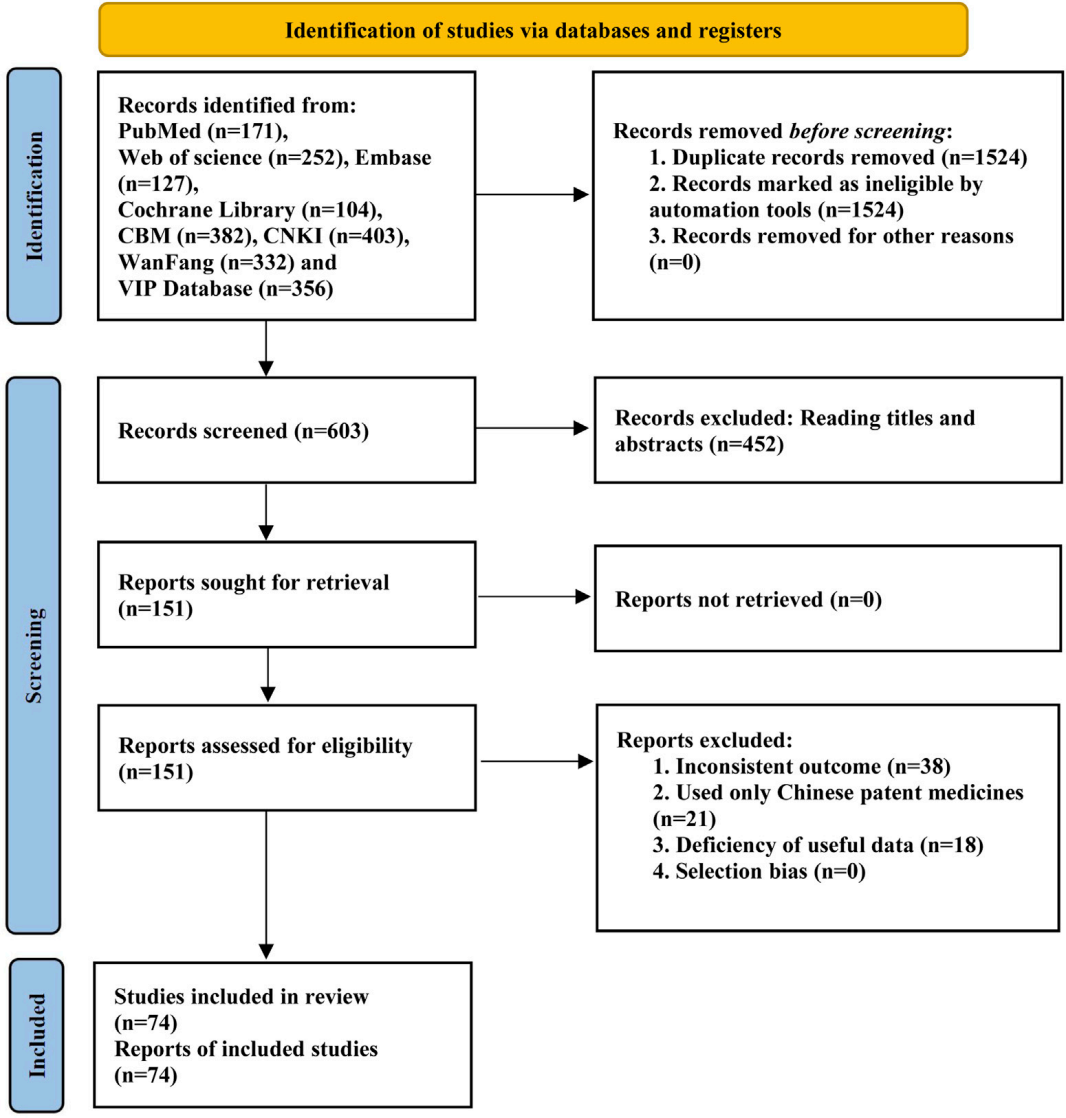
Flow chart of literature screening.

After the reading of the title, abstract and full text, a total of 74 RCTs were finally included (Wang, 2005; Song, 2011; Yang G., 2011; Yang R., 2011; Huang, 2012; Zhang, 2013; Lei, 2014; Wang He et al., 2015; Wang Namei et al., 2015; Sun and Fang, 2015; Wang and Hu, 2016; Song, 2016; Wang, 2016; Zhang et al., 2016; Alimu, 2017; Chen, 2017; Gong, 2017; Chen et al., 2017; Guo, 2017; Hao, 2017; Huang, 2017; Li C., 2017; Li Y., 2017; Sang, 2017; Wen, 2017; Yu, 2017; Cao, 2018; Hu and Liu, 2018; Hua and Dai, 2018; Cang et al., 2018; Wang J., 2018; Wang L., 2018; Zhao, 2018; Ban, 2019; Zhang, 2019; Deng, 2020; Lei, 2020; Li, 2020; Shang and Shang, 2020; Shi and Zhang, 2020; Wang, 2020; Liu and Feng, 2020; Xu, 2020; Zhang, 2020; Dong et al., 2021; Lei and Yang, 2021; Quan and Huang, 2021; Wan and Hu, 2021; Yang, 2021; Yin, 2021; Zeng, 2021; Chunyang and Song, 2022; Hu, 2022; Tong and Zhang, 2022; Li, 2022; Ma and Xu, 2022; Ma, 2022; Wang et al., 2022; Xiong, 2022; Xu, 2022; Xue, 2022; Yang, 2022; Yuqing and Yu, 2022; Zhao, 2022; Ji, 2023; Jia, 2023; Feng et al., 2023; Liu et al., 2023; Xiao and Chen, 2023; Xu, 2023; Xiao and Chen, 2023; Yu, 2023; Zeng, 2023; Zhang, 2023). The literature screening process is shown in Figure 1, and the basic information of the included studies is shown in Table 1.

**TABLE 1:** General characteristics of the included RCTs.

Study	Sample size		Age (years)		Interventions		Course (days)	Outcome indicator
	T	C	T	C	T	C		
Zeng (2021)	47	47	34.1 ± 5.88	32.6 ± 5.81	BFKS (1 capsule, *qd*) + CON	Enhancement period: Clotrimazole Vaginal tablets (0.5 g, once every 4 days)	30d	①②
					BFKS (1 *qd* for 7d, once every 7 days) + CON	Consolidation period: Clotrimazole Vaginal tablets (0.5 g, once every 15 days)	30d	
Wang L et al. (2018)	30	30	42.7 ± 1.8	43.1 ± 1.9	BFKS (1 capsule, *qd* for 8 days) + CON	*Lactobacillus* vaginalis capsules (2 capsules, *qd* for 7 days)	15d	①⑤
Hu and Dai (2018)	40	40	39.53 ± 8.72	36.4 ± 9.84	BFKS (1 capsule, *qd*) + CON	Fluconazole (Oral, first dose 0.4 g, then 0.2 g for 6 days, *qd*) + 4% sodium bicarbonate solution (Vulval and vaginal)	7d	①
Hao (2017)	44	44	28.9 ± 2.1	29.6 ± 2.3	BFKS (1 capsule, *qd*) + CON	Fluconazole capsules (Oral, 150 mg, once every 3 days)	7d	①
Li C et al. (2017)	45	45	27.38 ± 1.98	25.34 ± 2.78	BFKS (1 capsule, *qd*) + CON	5% sodium bicarbonate solution (Vulval and vaginal)	8d	①③④
Alimu, (2017)	100	100	34.59 ± 2.58	34.31 ± 2.7	BFKS (1 capsule, *qd*) + CON	*Lactobacillus* vaginalis capsules (2 capsules, *qd*)	7d	①③④
Wang and Hu (2016)	59	59	38.7 ± 5.2	39.1 ± 4.7	BFKS (1 capsule, *qd*) + CON	*Lactobacillus* vaginalis capsules (2 capsules, *qd*)	7d	①②⑤
Huang (2012)	103	91	37.2 ± 6.4	37.2 ± 6.4	BFKS (1 capsule, *qd*) + CON	Itraconazole capsules (Oral, 1 capsule, *bid*)	6d	①⑤
Yang et al. (2011a)	47	47	21–62	21–62	BFKS (1 capsule, *qd*) + CON	Ketoconazole (Oral, 200 mg, *bid*)	7d	①②
Wang (2005)	50	50	31.56 ± 4.6	31.56 ± 4.6	BFKS (1 capsule, *qd*) + CON	Spironolactone tablets (Oral, 200 mg, *bid*)	7d	①②⑤
Li (2020)	80	80	35.53 ± 6.29	37.32 ± 5.16	FFKS (3–5 mL, *qd*) + CON	Miconazole nitrate suppository (1 capsules, *qd*)	7d	①⑤
Xu (2020)	100	100	37.12 ± 1.34	37.12 ± 1.34	FFKS (3–5 mL, *qd*) + CON	Miconazole nitrate suppository (1 capsule, *qd*)	7d	①⑤
Zhang D et al. (2016)	33	31	18–50	18–50	FFSJZYS (1 capsule, *qd*) + CON	Fluconazole (Oral, 150 mg on the 1st and 4th days)	12d	①②⑤
Lei (2014)	23	23	20–57	19–55	FFSJZYS (1 capsule, *qd*) + CON	Miconazole nitrate suppository (1 capsule, *qd*)	7d	①
Yang (2011b)	90	90	42 ± 7.2	42 ± 7.2	FFSJZYS (1 capsule, *qd*) + CON	Miconazole nitrate suppository (1capsule, *qd*)	7d	①②
Xue (2022)	45	45	35.12 ± 6.11	35.24 ± 6.17	HHFJL (10 mL, *bid*) + CON	Metronidazole, Clotrimazole and Chlorhexidine Acetate Vaginal Effervescent tablets (1 tablet, *qd*)	7d	③④
Quan and Huang (2021)	98	98	43.6 ± 3.5	44.2 ± 3.3	HHFJL (10 mL, *bid*) +CON	Clotrimazole Vaginal tablets (1 tablet, *qd*)	7d	①②③④⑤
Zhang (2020)	43	43	32.4 ± 3.1	30.9 ± 3.4	HHFJL (10 mL, *bid*) + CON	Nystatin (Oral, 2 tablets, *tid*)	7d	⑤
Shi and Shang (2020)	135	135	35.65 ± 4.13	34.79 ± 4.25	HHFJL (10 mL, *bid*) + CON	Clotrimazole Vaginal tablets (0.5 g, once every 3 days)	7d	①②
Zhang (2019)	26	26	43.54 ± 5.27	42.56 ± 5.29	HHFJL (10 mL, *bid*) + CON	Clotrimazole Vaginal tablets (0.5 g, *qd*)	10d	①⑤
Ban (2019)	89	89	26.7 ± 2.8	26.7 ± 2.8	HHFJL (10 mL, *bid*) + CON	Nifuratel and Nysfungin Vaginal Soft Capsules (1 capsule, *qd*)	7d	①②⑤
Wang (2018a)	50	50	25.90 ± 3.44	25.85 ± 3.50	HHFJL (10 mL, *bid*) + CON	Miconazole nitrate suppository (200 mg, *qd*)	7d	⑤
Cang et al. (2018)	78	78	40.18 ± 5.26	39.74 ± 5.07	HHFJL (10 mL, *bid*) + CON	Clotrimazole Vaginal tablets (1 table, *qd*)	14d	①③④
Yu (2017)	21	21	29.0 ± 1.2	31.0 ± 1.5	HHFJL (10 mL, *bid*) + CON	Miconazole nitrate suppository (1 capsule, *qd*)	7d	①⑤
Li (2017b)	46	46	26.9 ± 3.2	26.2 ± 3.4	HHFJL (10 mL, *bid*) + CON	Miconazole nitrate suppository (200 mg, *qd*)	7d	①②
Wang et al. (2015b)	107	106	27.7	27.5	HHFJL (10 mL, *bid*) + CON	Nifuratel and Nystatin Vaginal Suppositories (1capsule, *qd*)	7d	①②
Sun and Fang (2015)	77	77	26.7 ± 2.7	26.7 ± 2.7	HHFJL (10 mL, *bid*) + CON	Miconazole nitrate suppository (200 mg, *qd*)	7d	①②
Chen et al. (2017)	60	60	25–48	25–48	JEYL (50 mL, *qd*) + CON	Clotrimazole Suppositories (0.15 g, *qd*)	7d	①②
Lei and Yang (2021)	46	46	37.78 ± 7.57	38.13 ± 8.25	KFG (1 pill, *qd*) + CON	Clotrimazole Suppositories (0.15 g, *qd*)	14d	①②③④
Wan and Hu (2021)	71	68	26.61 ± 3.66	27.37 ± 3.75	KFG (1 pill, *qd*) + CON	Nifuratel and Nystatin Vaginal Suppositories (1capsule, *qd*)	6d	①②
Ma and Xu (2022)	30	30	34.8 ± 2.7	34.6 ± 2.6	KSG (1 pill, *qd*) + CON	Fluconazole (Oral,150 mg, once every 3 days + microwave therapy machine for 20–30 min +4% NaHCO_3_)	14d	①②③④⑤
Yang (2021)	46	46	33.14 ± 2.08	32.41 ± 2.18	KSG (1 pill, *qd*) + CON	Nifuratel and Nystatin Vaginal Suppositories (1 capsule, *qd*)	12d	②③④⑤
Yin (2021)	40	40	28.3 ± 1.5	28.7 ± 1.3	KSG (1 pill, *qd*) + CON	Ketoconazole (Oral, 400 mg, *qd*)	30d	②③⑤
Shang and Shang (2020)	53	53	34.82 ± 10.26	34.52 ± 9.12	KSG (1 pill, *qd*) + CON	Fluconazole capsules (Oral, 1 capsule, once every 3 days for 4 times)	14d	①②
Deng (2020)	65	65	33.15 ± 3.16	32.98 ± 2.80	KSG (1 pill, *qd*) + CON	Fluconazole capsules (Oral, 0.15 g, *qd*)	8d	①②⑤
Liu and Feng (2020)	40	40	39.61 ± 5.14	39.69 ± 5.20	KSG (1 pill, *qd*) + CON	Metronidazole, Clotrimazole and Chlorhexidine Acetate Vagina Gel (1 pill, *qd*)	7d	①②③④⑤
Wang (2020)	40	40	28.4 ± 2.1	27.6 ± 2.3	KSG (1 pill, *qd*) + CON	Fluconazole capsules (Oral, 0.15 g, once every 3 days for 4 times)	14d	①②③④⑤
Cao (2018)	70	70	48.33 ± 3.47	47.89 ± 3.38	KSG (1 pill, *qd*) + CON	Fluconazole capsules (Oral, 150 mg, *qd*)	7d	①③④
Zhao (2018)	38	38	47.1 ± 3.4	46.7 ± 3.8	KSG (1 pill, *qd*) + CON	Fluconazole capsules (Oral, 150 mg + 2%–4% sodium bicarbonate solution, *qd*)	14d	①③④
Guo (2017)	210	190	42.01 ± 12.45	41.37 ± 12.66	KSG (1 pill, *qd*) + CON	Fluconazole capsules (Oral, 0.15 g, *qd*)	14d	①②
Wen (2017)	36	36	32.14 ± 1.32	32.2 ± 1.28	KSG (1 pill, *qd*) + CON	Fluconazole (Oral, 150 mg on the 1st and 4th days)	14d	①③④
Gong (2017)	66	66	34.9 ± 3.4	35.5 ± 3.3	KSG (1 pill, *qd*) + CON	Fluconazole capsules (Oral, 0.15 g, *qd*)	14d	①②③④
Sang (2017)	52	52	28.5 ± 5.6	27.4 ± 4.8	KSG (1 pill, *qd*) + CON	Fluconazole capsules (Oral, 0.15 g, once every 3 days for 4 times)	14d	①②③④
Huang (2017)	50	50	35.6 ± 3.5	36.1 ± 3.6	KSG (1 pill, *qd*) + CON	Fluconazole (Oral, 150 mg on the 1st and 4th days)	7d	①③④
Chen (2017)	45	45	38.23 ± 3.43	38.16 ± 3.47	KSG (1 pill, *qd*) + CON	Fluconazole (Oral, 150 mg on the 1st and 4th days)	7d	①③④
Song (2016)	53	53	33.6 ± 8.7	33.6 ± 8.7	KSG (1 pill, *qd*) + CON	Fluconazole capsules (Oral, 0.15 g, once every 3 days for 4 times)	14d	①②
Wang (2016)	42	41	32.5 ± 1.1	32.5 ± 1.1	KSG (1 pill, *qd*) + CON	Fluconazole capsules (Oral, 0.15 g, *tid*)	12d	①②③④
Wang et al. (2015b)	43	42	26.2 ± 2.9	26.2 ± 2.9	KSG (1 pill, *qd*) + CON	Fluconazole capsules (Oral, 0.15 g, once every 3 days for 4 times)	14d	①②③④
Zhang (2023)	40	40	35.41 ± 3.24	35.41 ± 3.24	BAL (10 mL, *bid*) +CON	Metronidazole, Clotrimazole and Chlorhexidine Acetate Vagina Gel (1 pill, *qd*)	30d	②③④⑤
Lei (2020)	33	33	31.01 ± 4.01	30.51 ± 3.81	BAL (10 mL, *bid*) + CON	Nifuratel and Nystatin Vaginal Suppositories (1 capsule, *qd*)	7d	①
Dong et al. (2021)	64	64	35.64 ± 2.38	34.76 ± 2.41	BAL (10 mL, *bid*) + CON	Nifuratel and Nystatin Vaginal Suppositories (1 capsule, *qd*)	7d	①③④⑤
Yang (2022)	20	20	42.88 ± 3.93	42.91 ± 3.92	BFKS (1 capsule, *qd*) + CON	Clotrimazole Suppositories (1 capsule, *qd*)	30d	①②③④⑤
Xu (2023)	30	30	27.97 ± 5.52	27.82 ± 5.48	BFKS (1 capsule, *qd* for 8 days) + CON	Lacidophilin Vaginal Capsules (2 capsules, *qd*)	15d	①④⑤
Hua and Dai, (2018)	30	30	31.1 ± 4.1	30.6 ± 4.3	BFKS (1 capsule, *qd*) + CON	Miconazole Nitrate Suppositories (1 capsule, *qd*)	7d	①⑤
Tong and Zhang (2022)	35	35	35.2 ± 1.5	37.1 ± 1.4	BFKS (1 capsule, *qd*) + CON	Fluconazole capsules (Oral, 0.15 g, once every 2 days for 3 times)	30d	①②⑤
Hu (2022)	45	45	28.65 ± 2.31	29.45 ± 2.33	BFKS (1 capsule, *qd*) + CON	Lacidophilin Vaginal Capsules (2 capsules, *qd*)	15d	①③④
Xiong (2022)	50	50	33.50 ± 3.75	33.65 ± 3.65	FFKS (3–5 mL, *qd*) + CON	Clotrimazole Suppositories (1 capsule, *qd*)	28d	①⑤
Zhang (2013)	98	98	28.46 ± 3.31	28.46 ± 3.31	FFKS (3–5 mL for mild cases, 20–50 mL for severe cases, *qd*) + CON	Fluconazole capsules (Oral, 0.15 g, *qd*)	7d	①
Jia (2023)	40	40	35.26 ± 1.57	35.42 ± 1.75	HHFJL (10 mL, *bid*) + CON	Clotrimazole Vaginal Tablets (1 table, *qd*)	10d	①②③④
Xiao and Chen (2023)	30	30	33.41 ± 3.77	33.27 ± 3.70	HHFJL (50 mL, *qd*) + CON	Fluconazole capsules (Oral, 0.15 g, once every 2 days for 14 times)	28d	①②③④⑤
Zhao et al. (2022)	47	47	38.41 ± 6.38	38.65 ± 5.49	HHFJL (10 mL, *bid*) + CON	Lacidophilin Vaginal Capsules (2 capsules, *qd*)	7d	①
Liu et al. (2023)	40	40	42.92 ± 6.53	42.87 ± 6.46	HHFJL (10 mL, *bid*) + CON	Nifuratel and Nystatin Vaginal Suppositories (1 capsule, *qd*)	7d	①②③④⑤
Yu (2023)	28	28	36.98 ± 3.54	37.04 ± 3.61	HHFJL (10 mL, *bid*) + CON	Metronidazole, clotrimazole and Chlorhexidine Acetate Vaginal Effervescent tablets (1 table, *qd*)	—	①③④
Song (2011)	39	24	21–48	21–48	JEYL (50 mL, *qd*) +CON	Nystatin (2 pills, *qd*)	14d	①⑤
Wang at al. (2022)	51	51	38.9 ± 5.2	38.7 ± 5.3	KFG (1 pill, *qd*) + CON	Clotrimazole cream (10 g, *qd*)	7d	①②⑤
Li (2022)	45	45	33.92 ± 5.69	34.79 ± 4.95	KFG (1 pill, *qd*) + CON	Clotrimazole Suppositories (1 capsule, *qd*)	14d	①②③④
Feng et al. (2023)	50	50	42.7 ± 5.2	42.9 ± 5.3	KSG (1 pill, *qd*) + CON	Fluconazole tablets (150 mg at once, another one after 3d for severe cases)	14d	①②③④⑤
Li (2022)	49	49	46.3 ± 5.34	43.5 ± 1.34	KSG (1 pill, *qd*) + CON	Clotrimazole Vaginal Tablets (0.15 g, once every 2 days for 7 times)	21d	①③④⑤
Yuqing and Yu, 2022	37	37	34.86 ± 4.41	34.92 ± 4.58	KSG (1 pill, *qd*) + CON	Clotrimazole Suppositories vaginal (1 piece on the 1st, 5th and 8th days)	21–28d	①②③④⑤
Ji (2023)	100	100	32.4 ± 25.4	33.2 ± 2.3	KSG (1 pill, *qd*) + CON	Fluconazole capsules (Oral, 0.15 g, *qd*)	14d	①②③④⑤
Ma (2022)	40	40	34.88 ± 3.45	34.89 ± 3.42	KSG (1 pill, *qd*) + CON	Fluconazole capsules (Oral, 0.15g, *qd*)	14d	①②③④⑤
Pi (2023)	55	55	29.65 ± 7.10	29.76 ± 7.04	KSG (1 pill, *qd*) + CON	Fluconazole capsules (Oral, 0.15 g, once every 3 days for 4 times)	14d	①③④
Zeng (2023)	75	75	27.61 ± 3.06	27.52 ± 3.34	KSG (1 pill, once every 2 days for 7 times) + CON	Policresulen Vaginal Suppositories (1 piece, once every 2 days for 7 times)	15d	①②③④
Xu (2022)	60	60	41.85 ± 9.64	42.37 ± 9.52	KSG (1 pill, *qd*) + CON	Metronidazole, Clotrimazole and Chlorhexidine Acetate Vagina Gel (1pill, *qd*)	14d	①②⑤

Note: T, test group; C, control group; CON, intervention measures in the control group; *qd*, Once every day; *bid*, Twice every day; *tid*, Three times every day; ① negative conversion rate of C. albicans; ② recurrence rate of C. albicans; ③ time to resolution of pruritus; ④ time to resolution of secretion; ⑤ occurrence of adverse reactions.

A total of 8,151 patients were involved: KSG (26 RCTs), HHFJL (17 RCTs), 26 BFKS (15 RCTs), KFG (4 RCTs), FFKS (4 RCTs), BAL (3 RCTs), FFSJZYS (3 RCTs), JEYL (2 RCTs). The number of sample sizes in the included literature ranges from 20 to 210, with an average of 55. The range of age is from 23 to 51, with a mean age of 34. The course of treatment ranges from 6 d to 30d, and around 7d or 14d for most studies.

### 3.2 Quality evaluation of the included articles

**FIGURE 2:**
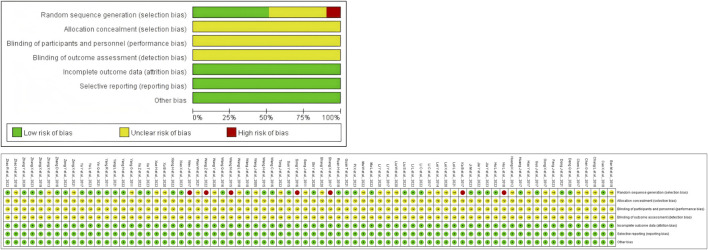
Percentages of items of included articles that produced risks of bias.

All studies were RCTs, of which 35 used random number table; three used random drawing; seven used the order of participation, the remaining studies only mentioned randomization. None of the studies mentioned blinding and allocation concealment. All studies had complete data. The risk of bias assessment of the included studies is shown in Figure 2.

### 3.3 Negative conversion rate of C. albicans

**FIGURE 3:**
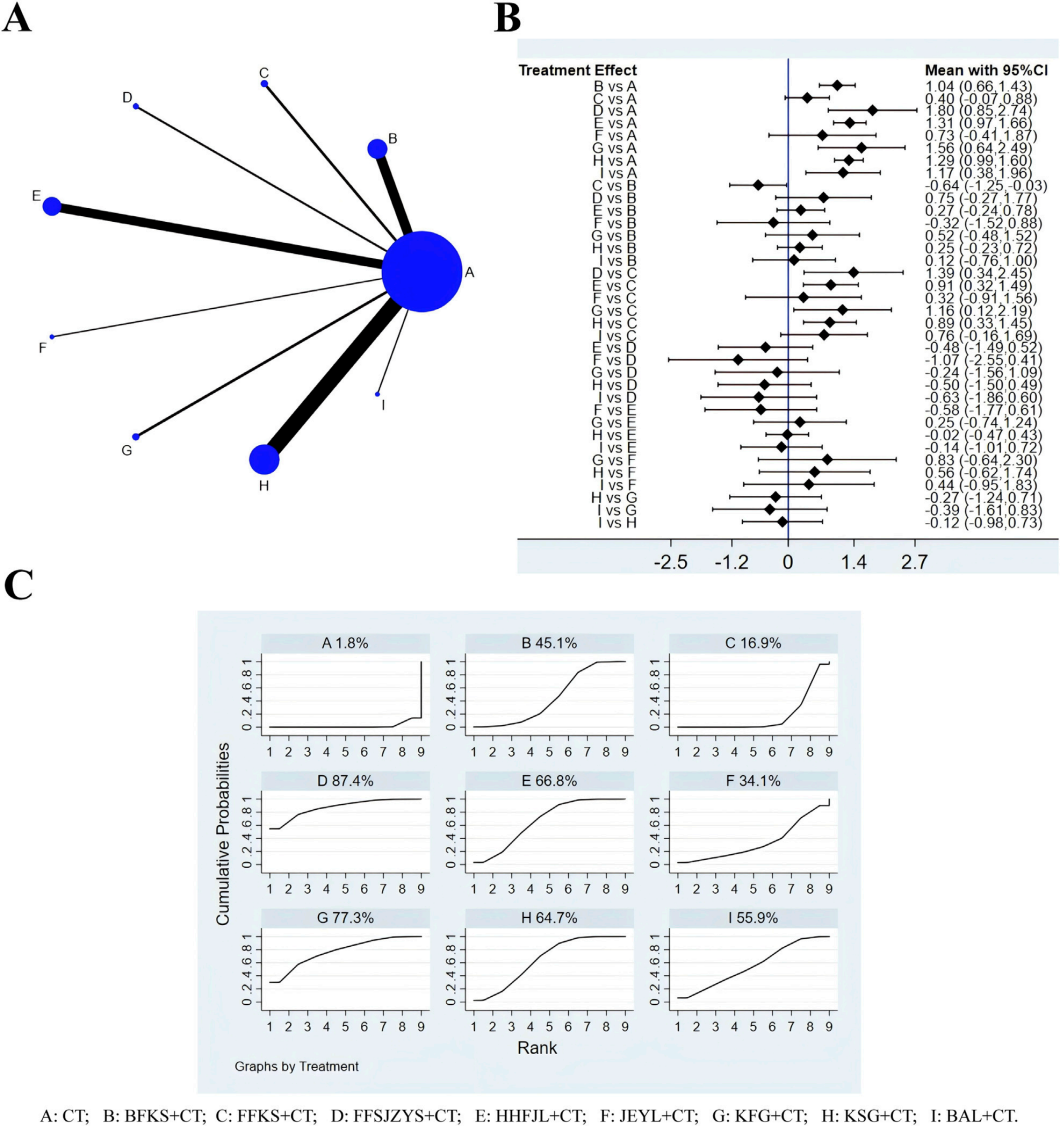
Evidence relationship **(A)**, forest plot **(B)** and SUCRA **(C)** of negative conversion rate of C. albicans. CT, conventional treatment; BFKS, Baofukang suppository; FFKS, Fufukang spray; FFSJZYS, Fufang Shajiziyou suppository; HHFJL, Honghe Fujie lotion; JEYL, Jieeryin lotion; KFG, Kangfu gel; KSG, Kushen gel; BAL, Bai’an lotion.

A total of 68 RCTs reported negative conversion rate of C. albicans, all nine interventions were involved. It was found that the overall I^2^ between the two-arm studies was 0.0%, indicating that the heterogeneity was small, and thus the fixed-effects model was used for NMA. As shown in Figure 3A, the larger the circle in the graph, the more patients in this measure; the thicker the line, the more studies in which directly the two interventions were directly compared. The results of NMA (Figure 3B) showed that compared with CT, CT combined with JEYL (OR = 0.73, 95% CI [-0.41, 1.87]) and FFKS (OR = 0.40, 95% CI [-0.07, 0.88]) could not significantly improve the conversion rate, but the rest of the test groups could significantly improve the negative conversion rate (*P* < 0.05). The analysis among test groups showed that compared with CT combined with FFKS, CT combined with FFSJZYS, BFKS, KSG, HHFJL, KFG or BAL could significantly improve the negative conversion rate (*P* < 0.05). The ranking results of each intervention measure were shown in Figure 3C. The percentage represented the surface under the cumulative ranking curve (SUCRA). The larger the area, the higher the efficiency. The results showed that the ranking of the nine interventions in improving the negative conversion rate was FFSJZYS + CT > HHFJL + CT > KFG + CT > KSG + CT > BAL + CT > BFKS + CT > JEYL + CT > FFKS + CT > CT.

### 3.4 Recurrence rate of C. albicans

**FIGURE 4:**
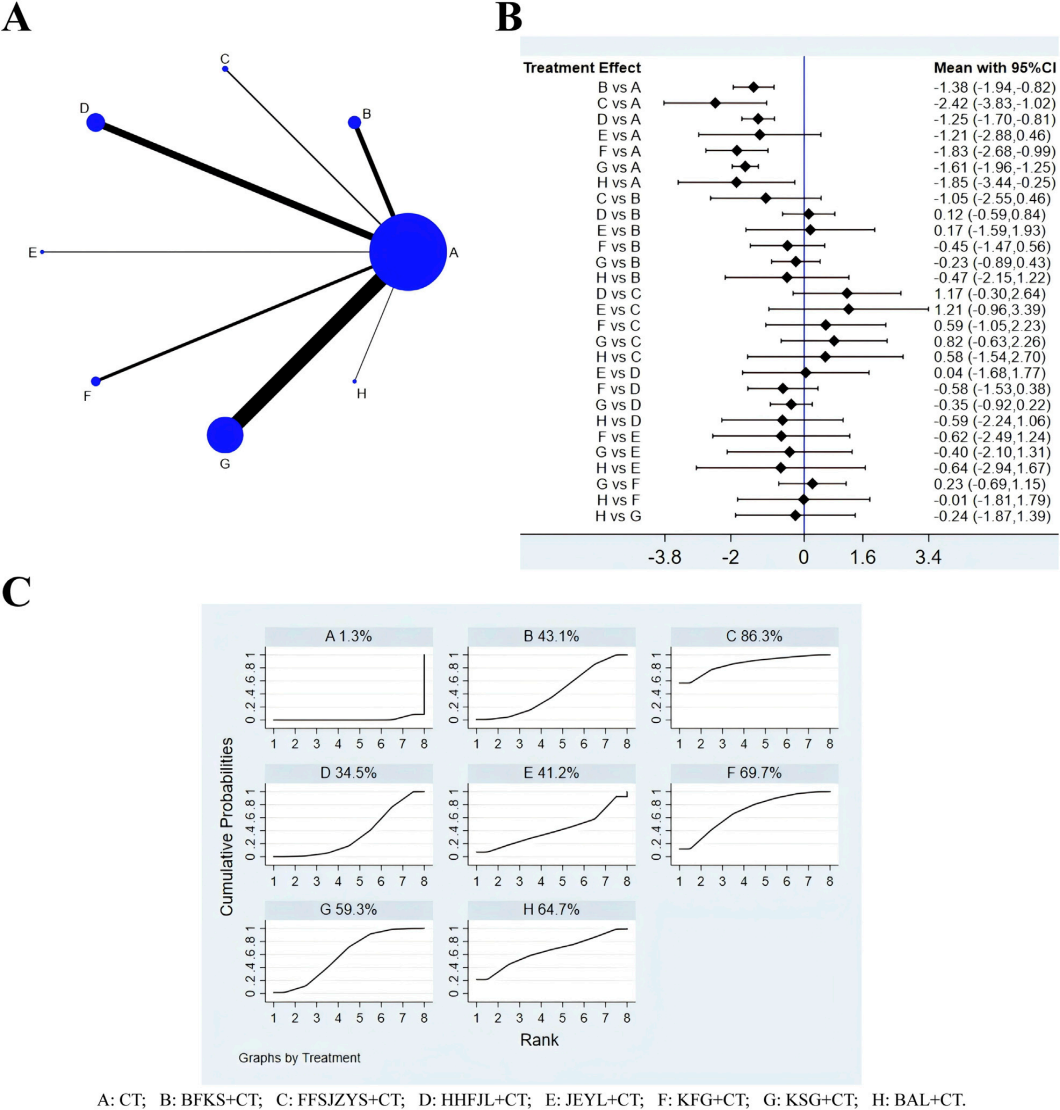
Evidence relationship **(A)**, forest plot **(B)** and SUCRA **(C)** of recurrence rate. CT, conventional treatment; BFKS, Baofukang suppository; FFSJZYS, Fufang Shajiziyou suppository; HHFJL, Honghe Fujie lotion; JEYL, Jieeryin lotion; KFG, Kangfu gel; KSG, Kushen gel; BAL, Bai’an lotion.

According to the evidence network (Figure 4A), a total of 42 studies reported recurrence rates, excluding FFKS, the remaining eight intervention measures were involved. Because the overall I^2^ between the two-arm studies was 0.0%, the fixed-effects model was used for NMA. The results of NMA (Figure 4B) showed that compared with CT, CT combined with JEYL (OR = −1.21, 95% CI [-2.88, 0.46]) could not significantly reduce the recurrence rate (*P* > 0.05), but the other test groups were significantly different from the CT groups (*P* < 0.05). Comparisons were made between the test groups, there was no significant difference. The SUCRA probability ranking of eight interventions (Figure 4C) in reducing the recurrence rate was FFSJZYS + CT > KFG + CT > BAL + CT > KSG + CT > BFKS + CT > JEYL + CT > HHFJL + CT > CT.

### 3.5 Time to resolution of pruritus

**FIGURE 5:**
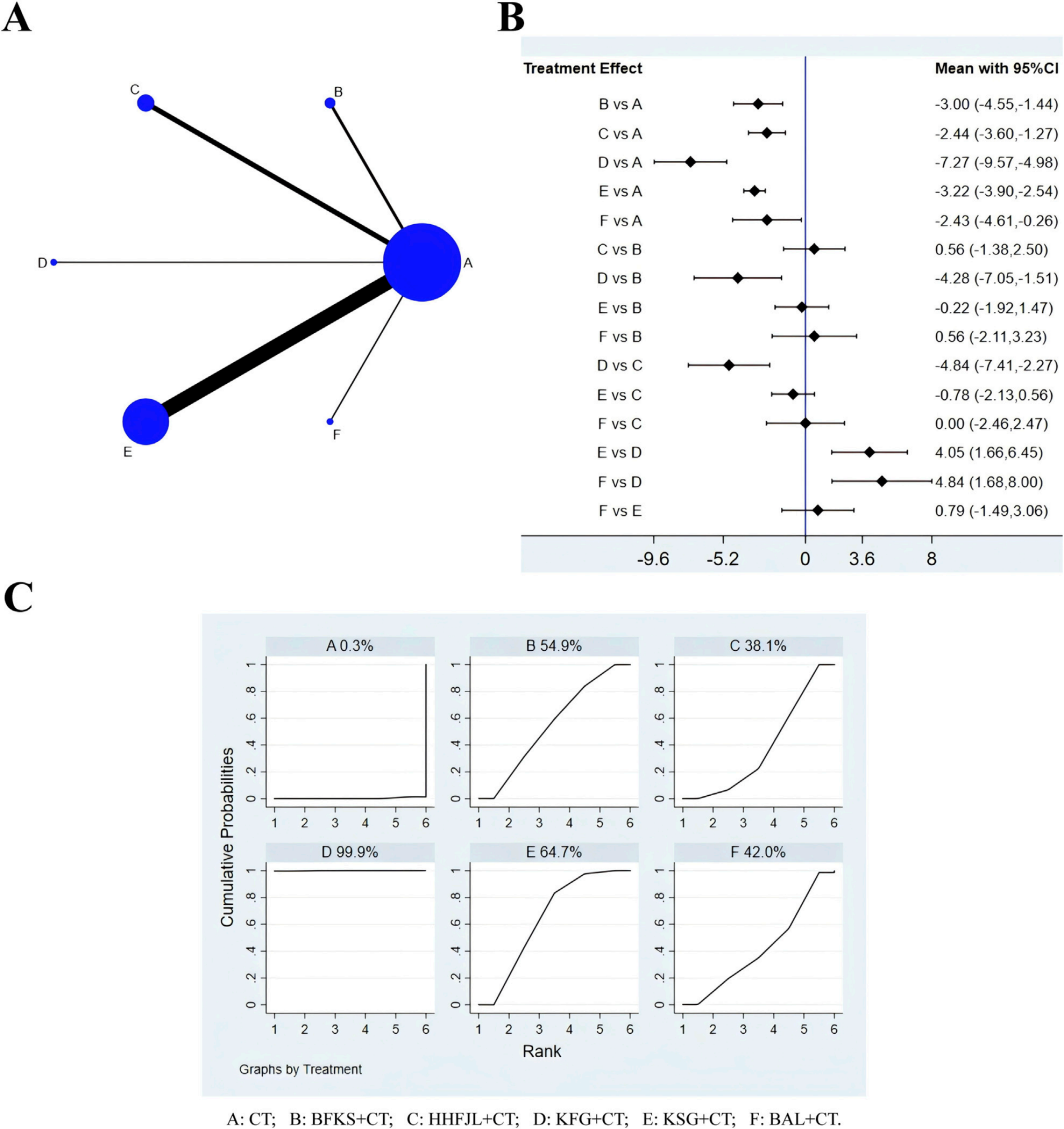
Evidence relationship **(A)**, forest plot **(B)** and SUCRA **(C)** of time to resolution of pruritus. CT, conventional treatment; BFKS, Baofukang suppository; HHFJL, Honghe Fujie lotion; KFG, Kangfu gel; KSG, Kushen gel; BAL, Bai’an lotion.

There were 36 studies whose outcome measures included time to resolution of pruritus, including six interventions: CT, BFKS, KSG, HHFJL, KFG, and BAL (Figure 5A). The results showed that the overall I^2^ between the two-arm studies was 94.0%, indicating that the heterogeneity was large, and thus the random-effects model was used for NMA. The results of NMA (Figure 5B) showed that compared with CT, CT combined with KFG (SMD = −7.27, 95%CI [-9.57, −4.98]), KSG (SMD = −3.22, 95%CI [-3.90, −2.54]), BFKS (SMD = −3.00, 95%CI [-4.55, −1.44]), BAL (SMD = −2.43, 95%CI [-4.61, −0.26]), or HHFJL (SMD = −2.44, 95%CI [-3.60, −1.27]) significantly shortened the time to resolution of pruritus (*P* < 0.05). When comparisons were made between the test groups, compared with CT combined with KSG (SMD = −4.05, 95%CI [-6.45, −1.66]), BFKS (SMD = −4.28, 95%CI [-7.05, −1.51]), BAL (SMD = −4.84, 95%CI [-8.00, −1.68]), HHFJL (SMD = −4.84, 95%CI [-7.41, −2.27]), CT combined with KFG could reduce pruritus more effectively (*P* < 0.05). The ranking of SUCRA (Figure 5C) probability was KFG + CT > KSG + CT > BFKS + CT > BAL + CT > HHFJL + CT > CT.

### 3.6 Time to resolution of secretion

**FIGURE 6:**
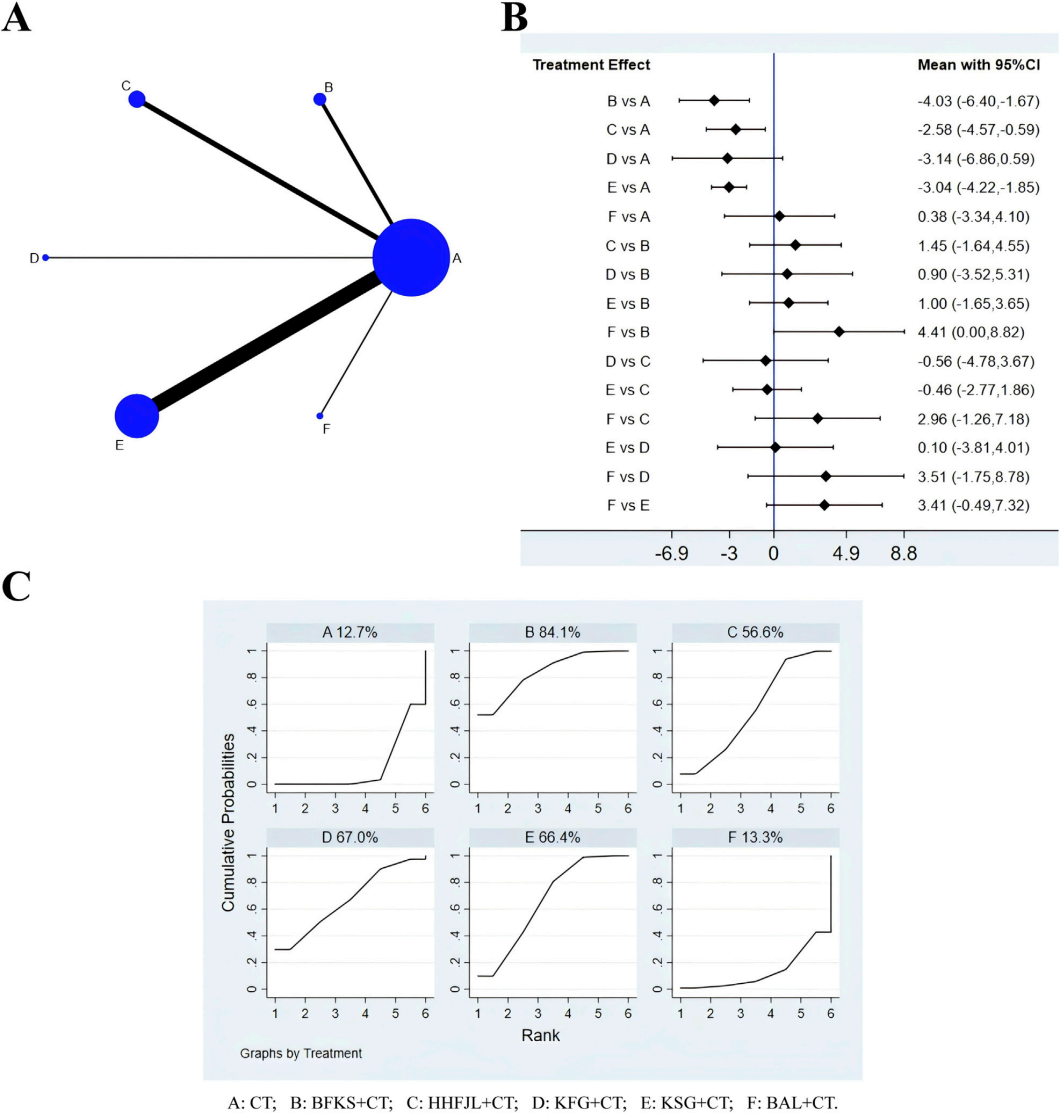
Evidence relationship **(A)**, forest plot **(B)** and SUCRA **(C)** of time to resolution of secretion. CT, conventional treatment; BFKS, Baofukang suppository; HHFJL, Honghe Fujie lotion; KFG, Kangfu gel; KSG, Kushen gel; BAL, Bai’an lotion.

A total of 36 studies reported time to resolution of secretion, including six interventions (Figure 6A): CT, BFKS, KSG, HHFJL, KFG, and BAL. Because the overall I^2^ was 97.3%, so the random-effects model was used for analysis. The results (Figure 6B) showed that compared with CT, CT combined with BFKS (SMD = −4.03, 95% CI [-6.40, −1.67]), KSG (SMD = −3.04, 95% CI [-4.22, −1.85]), or HHFJL (SMD = −2.58, 95% CI [-4.57, - 0.59]) could reduce abnormal leucorrhea more quickly (*P* < 0.05), but there was no significant difference between the test groups. The ranking of SUCRA probability in decreasing time to resolution of secretion was BFKS + CT > KFG + CT > KSG + CT > HHFJL + CT > BAL + CT > CT (Figure 6C).

### 3.7 Adverse reaction

**FIGURE 7:**
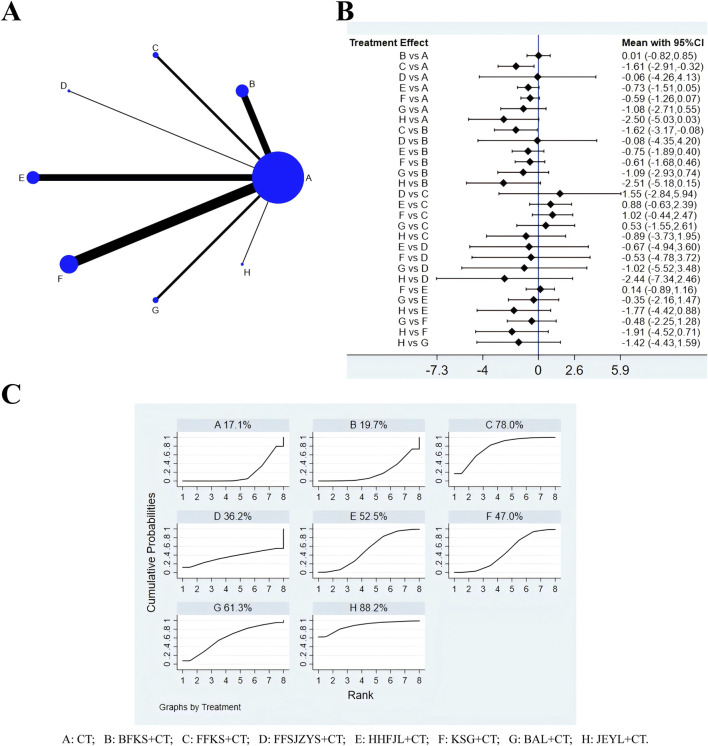
Evidence relationship **(A)**, forest plot **(B)** and SUCRA **(C)** of adverse reactions. CT, conventional treatment; BFKS, Baofukang suppository; FFKS, Fufukang spray; FFSJZYS, Fufang Shajiziyou suppository; HHFJL, Honghe Fujie lotion; KSG, Kushen gel; BAL, Bai’an lotion; JEYL, Jieeryin lotion.

A total of 36 studies reported adverse reactions, including eight interventions (Figure 7A): CT, BFKS, KSG, HHFJL, FFKS, FFSJZYS, BAL, and JEYL. Because the overall I^2^ was 0.0%, the fixed-effects model was used for NMA. The results of this analysis (Figure 7B) suggested that compared with CT, CT combined with FFKS (OR = −1.61, 95% CI [-2.91, −0.32]) had fewer side effects (*P* < 0.05). Compared with CT combined with BFKS, CT combined with FFKS (SMD = −1.62, 95% CI [-3.17, −0.08]) could significantly reduce adverse reactions (*P* < 0.05). The ranking of SUCRA probability in reducing adverse reactions was JEYL + CT > FFKS + CT > BAL + CT > HHFJL + CT > KSG + CT > FFSJZYS + CT > BFKS + CT > CT (Figure 7C).

### 3.8 Publication bias analysis

**FIGURE 8:**
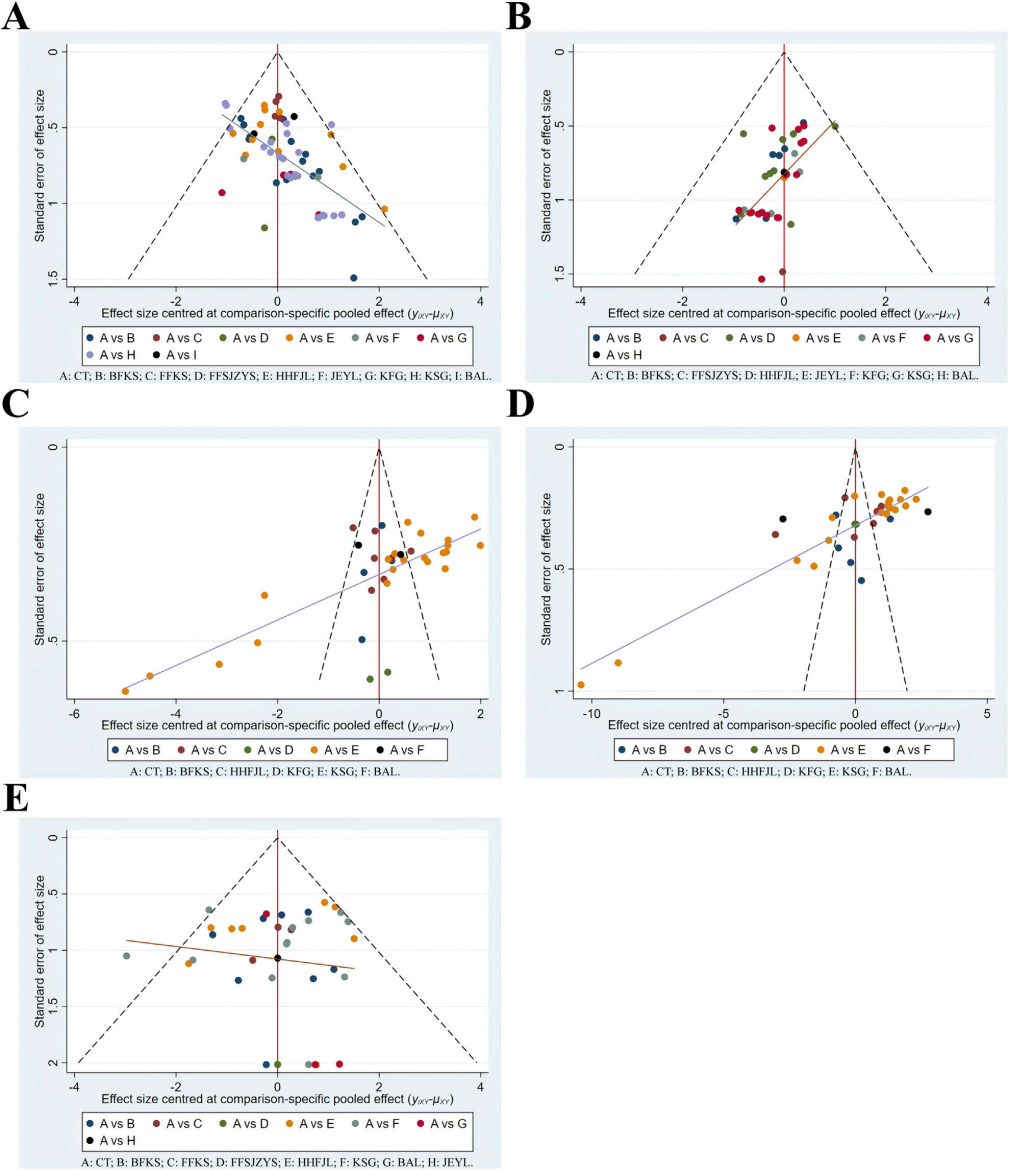
Funnel plots. **(A)** negative conversion rate; **(B)** recurrence rate; **(C)** time to resolution of pruritus; **(D)** time to resolution of secretion; **(E)** adverse reaction. CT, conventional treatment; BFKS, Baofukang suppository; FFKS, Fufukang spray; FFSJZYS, Fufang Shajiziyou suppository; HHFJL, Honghe Fujie lotion; JEYL, Jieeryin lotion; KFG, Kangfu gel; KSG, Kushen gel; BAL, Bai’an lotion.

With the effect size of each indicator as the abscissa and the standard error as the ordinate, a comparison-corrected funnel plot was drawn. Each dot represents a direct comparison of different interventions, and the number of dots in the same color represents the number of pairwise comparisons in the study. The results (Figure 8) demonstrated that the scattering points related to negative conversion rate, recurrence rate, and adverse reaction were basically within the range of the inverted funnel plot, and the graph was basically symmetrical about the zero line, indicating that the possibility of publication bias was small. Some scattering points involving time to resolution of pruritus and secretion were outside the range of the inverted funnel plot, suggesting that there was a high possibility of publication bias.

### 3.9 Inconsistency, subgroup and sensitivity analysis

Because five outcomes in this NMA were non-closed loops, the consistency hypothesis was not applicable in this study. Analyses revealed heterogeneity in the time to resolve pruritus and secretion, however, results of subgroup analyses based on age, course of disease, sample size, and course of treatment showed the heterogeneity did not been decreased (Supplementary Figure S1, S2). Sensitivity analyses were performed on the negative conversion rate of C. albicans, recurrence rate, time to resolution of pruritus, time to resolution of secretion, and incidence of adverse reactions. The results indicated that the sensitivity analyses for all outcomes were stable, with no significant bias detected, thereby affirming the stability of the meta-analysis results (Supplementary Figure S3).

## 4 Discussion

In the study, NMA was used for the first time to compare the efficacy of eight commonly used CCPPs for VVC. The analysis suggested that CT combined with FFSJZYS had better effect in both increasing the negative conversion rate and lowering recurrence rate of C. albicans. The underlying pharmacological mechanism may be related to its composition. FFSJZYS contains seven botanical drugs. Matrine and Osthole has been proved to reduce yeast to hypha transition of C. albicans (Shao et al., 2014; Li et al., 2017). *Boswellia sacra* Flück. [Burseraceae, *Olibanum*] and *Commiphora myrrha* (T.Nees) Engl. [Burseraceae, *Myrrha*] activate blood circulation and relieve pain, *Smithsonite* [*Calamina*] and *Dryobalanops aromatica* C.F.Gaertn [Dipterocarpaceae, *Borneolum*]. astringe sores and promote granulation. Most importantly, *Hippophae rhamnoides* L. [Elaeagnaceae, *Seabuckthorn Seed Oil*] is rich in carbohydrates, lipids and isoflavones (Zuchowski, 2023). Carbohydrates can provide nutrients for the growth of normal *lactobacillus* and lower vaginal pH; lipids aid in repairing vaginal tissues; and isoflavones can stimulate vaginal epithelial hyperplasia and keratosis. Taken together, FFSJZYS effectively addresses the heat, dampness, blood stasis and deficiency associated with VVC pathogenesis. Modern pharmacological studies have shown that FFSJZYS can regulate the local immune function of vaginal mucosa, repair the damaged epithelium, enhance the body resistance and improve the long-term prognosis of patients (Niu. et al., 2017; Zhao. et al., 2019).

In terms of improving pruritus, CT combined with KFG was more effective. KFG is composed of five botanical drugs. The extracts of *Angelica dahurica* (Hoffm.) Benth. and Hook.f. ex Franch. and Sav. [Apiaceae, *Angelicae Dahuricae Radix*] may exert analgesic effect by suppressing TRPV1 channel and reducing NO level (Li and Wu, 2017). *Zanthoxylum bungeanum* Maxim. [Rutaceae, *Zanthoxyli Pericarpium*] disperses cold and removes dampness, its volatile oil had the antipruritic effect *via* the GRPR pathway (Zhou et al., 2022). The total sesquiterpene lactones isolated from *Inula helenium* L. [Asteraceae, *Inulae Radix*] reduced epidermis/dermis thickening and dermal inflammatory infiltration (Wang et al., 2018). *Borneolum* can induce a cooling sensation and has significant antipruritic effects in the chronic pruritus models (Yin, 2019). In addition, the gel also has a lubricating and protective function. In summary, KFG has the effects of warming Yang and drying dampness, dispelling wind and relieving itching, has been shown to reduce the level of inflammatory factors such as IL-6, reduce the pruritus, pain and mucosal congestion of vulvovagina (Lei and Wang, 2021; Chunyang LI and Song, 2022).

In terms of resolving secretion, CT combined with BFKS ranked first. BFKS contains 8.2 mg *Curcuma zedoaria* (Christm.) Rosc. [Zingiberaceae, *Zedoary Turmeric oil*] and 7.5 mg *borneolum*. *Zedoary turmeric oil* can promote the regeneration of damaged mucosa and reduce inflammatory secretions (Kamazeri et al., 2012). *Borneolum* can also accelerate percutaneous drug absorption and improve the bioavailability of *zedoary turmeric oil* in vagina. Studies have shown that BFKS promotes the repair of vaginal epithelial cells, reduce IL-2, increase the cytokine IL-4, and upregulate the level of IgG secreted by the treated VK2/E6E7 cells (Li et al., 2016). The adverse reactions mainly involved vaginal dryness or heat, aggravated itching, rash, nausea, etc., which could be alleviated by stopping treatment. CT combined with FFKS, which includes *Rostellularia procumbens* (L.) Nees [Acanthaceae, *Rostellulariae procumbentis herba*], *Senecio scandens* Buch.-Ham. ex D.Don [Acanthaceae, S*enecionis scandentis herba*], has fewer adverse effects and can be used for pregnant patients (Duan et al., 2023). In addition, JEYL, BAL and HHFJL also rank high on reducing adverse reactions, it may be also related to liquid preparations that can be metabolized faster and are milder than suppositories. However, the difference is not statistically significant, it may be associate with the small sample size.

In addition, some mechanisms of other CCPPs in treating VVC have been studied. HHFJL is made from dry distillation liquid of *Crataegus pinnatifida* Bunge. [Rosaceae, *Crataegus Semen*], it could inhibit C. albicans biofilm formation (JIN et al., 2017), a meta-analysis indicates improvement of effectiveness and safety of combination medication with HHFJL in VVC, especially among pregnant women (Lijuan et al., 2022). KSG are composed of *Sophora flavescens* Aiton. [Fabaceae, Total Alkaloids of *Sophorae flavescentis Radix*], the results of meta-analysis by Du et al. showed that KSG could increase the negative conversion rate of pathogenic bacteria, reduce the recurrence rate and clinical symptoms, which was superior to that of antifungal drug alone (Du. et al., 2021). JEYL contains key components such as matrine, osthole, baicalein, quercetin, a study confirmed that the synergistic effect of baicalein and quercetin could effectively inhibit the adherence of *Candida* cells by down-regulating ALS3, HYR1 and so on (Janeczko et al., 2022). *Phellodendron chinense* C.K.Schneid. [Rutaceae, *Phellodendri Chinensis Cortex*] is the sovereign drug in the BAL. Modern studies show that Berberine can inhibit biofilm formation and the adhesion of C. albicans to vaginal epithelial cells by decreasing the ICAM-1 and mucin1 expressions, balancing IL-2 and IL-4 expressions (Huang et al., 2020; Zhao et al., 2022).

To sum up, considering the clinical implications and recommendations, FFSJZYS may be the preferred treatment for patients with serious imbalance in the vaginal micro-ecological environment or those who are not cured for a long time and often relapse. KFG demonstrates strong anti-itch properties, and may be a good choice for VVC patients with severe pruritus. BFKS may be a preferred choice when it comes to reducing leukorrhea. In the incidence of adverse reactions, FFKS may have more advantages. For pregnant women, HHFJL and FFKS are safer and clinically proven. Since JEYL is contraindicated, FFSJZYS and KSG should be used with caution according to the instructions; TCM advises against *borneolum* (in KFG and BAL) and warns that blood-activating drugs (in BFK and FFSJZYS) are risky during pregnancy. Finally, in terms of daily cost, lotions like BAL are more economical, while suppositories and gels are pricier, with BFKS being the more affordable option among them. The above discussions were made from the TCM theory, pharmacological research and clinical experience in gynecology, but it is still crucial to choose the appropriate CCPPs based on the results of this study and the actual situation of patients in clinical practice.

This study has the following shortcomings: 1) The quality of the included studies needs to be improved, because the studies were all conducted in China, high-quality English literature is lacking, and none of them mentioned blinding and allocation concealment, so there might be bias. 2) There are differences in the number of studies and sample sizes included for each CCPP, this increases the difference in the test efficacy. There is a lack of RCTs with pairwise comparison among different CCPPs, and the evidence relationship fails to form a closed loop, these can affect the reliability and stability of the conclusions to a certain extent. 3) There was some heterogeneity in the reduction of pruritus and secretion, but the subgroup network meta-analysis of age, course of disease, sample size, and duration of treatment showed that the heterogeneity did not decrease, which may be related to many interventions and differences in patients’ subjective consciousness, etc.

In view of the limitations, the recommendations are as follows: 1) Large-sample and multi-center RCTs of international cooperation should be organized, the research plan should be registered in advance and blinding and allocation concealment should be implemented strictly to reduce risk of bias (Yue et al., 2024). 2) the quality and quantity of articles included in meta-analysis should be increased, and the baseline differences should be controlled to avoid excessive heterogeneity that affects the stability and generalizability of the results; 3) for CCPPs with limited evidence (e.g., BAL), more clinical studies are needed to further verify their efficacy, and more RCTs for direct comparison between CCPPs can be conducted to improve the level of indirect comparative evidence. 4) The treatment of VVC is challenging, future research should undertake comprehensive analyses to investigate the impact of variables such as dosage, dosage form on treatment. This will help further offer better guidance.

## 5 Conclusion

The NMA showed that addition of CCPPs to CT may acquire a better curative effect in patients with VVC. FFSJZYS combined with CT is more effective in improving the negative conversion rate and controlling the recurrence rate of C. albicans. KFG combined with CT had the better effect in relieving pruritus. BFKS combined with CT were the most effective in reducing leukorrhea. FFKS combined with CT had fewer adverse reactions. The conclusions aforementioned are limited to the data analysis of literature, and cannot fully explain the clinical efficacy of CCPPs, larger sample sizes and more quality RCTs are needed in future.

## Data Availability

The original contributions presented in the study are included in the article/Supplementary Material, further inquiries can be directed to the corresponding authors.
