# Supplementary material for: Comparative efficacy of topical commercial Chinese polyherbal preparation for vulvovaginal candidiasis: a network meta-analysis
Source: Front Pharmacol. 2025 Feb 3;16:1484325. doi: 10.3389/fphar.2025.1484325 (PMC11830678; doi:10.3389/fphar.2025.1484325)
Supplement: Supplementary file 1 [file Table1.docx]

**Table S1. Composition of polyherbal preparations**

| **Drug name** | **Botanical drug name** |
| --- | --- |
| Baofukang suppository (BFKS) | *Curcuma zedoaria* (Christm.) Rosc. [Zingiberaceae, Zedoary Turmeric oil],  *Dryobalanops aromatica* C.F.Gaertn. [[Dipterocarpaceae](https://powo.science.kew.org/taxon/urn:lsid:ipni.org:names:77126600-1), *Borneolum*] |
| Fufukang spray (FFKS) | *Rostellularia procumbens* (L.) Nees [Acanthaceae, *Rostellulariae procumbentis herba*],  *Senecio scandens* Buch.-Ham. ex D.Don [Acanthaceae, S*enecionis scandentis* *herba*] |
|  |  |
| Fufang Shajiziyou suppository (FFSJZYS) | *Cnidium monnieri* (L.) Cusson. [Apiaceae, *Cnidii Fructus*],  *Boswellia sacra* Flück. [Burseraceae, *Olibanum*],  *Commiphora myrrha* (T.Nees) Engl. [Burseraceae, *Myrrha*],  *Sophora flavescens* Aiton. [[Fabaceae](https://powo.science.kew.org/taxon/urn:lsid:ipni.org:names:30000147-2), *Sophorae flavescentis Radix*], *Smithsonite* [*Calamina*],  *Dryobalanops aromatica* C.F.Gaertn. [[Dipterocarpaceae](https://powo.science.kew.org/taxon/urn:lsid:ipni.org:names:77126600-1), *Borneolum*],  *Hippophae rhamnoides* L. [Elaeagnaceae, *Seabuckthorn Seed Oil*] |
|  |  |
|  |  |
|  |  |
|  |  |
|  |  |
|  |  |
| Honghe Fujie lotion (HHFJL) | *Crataegus pinnatifida* Bunge. [Rosaceae, *Crataegus Semen*] |
| Jieeryin lotion (JEYL) | *Cnidium monnieri* (L.) Cusson. [Apiaceae, *Cnidii Fructus*],  *Artemisia argyi* H.Lév. & Vaniot. [Asteraceae, *Artemisiae Argyi Folium*],  *Angelica biserrata* (R.H.Shan & C.Q.Yuan) C.Q.Yuan & R.H.Shan [Apiaceae, *Angelicae Pubescentis Radix*],  *Acorus verus* (L.) Raf. [Araceae, *Acori Tatarinowii Rhizoma*],  *Atractylodes lancea* (Thunb.) DC. [Asteraceae, *Atractylodis Rhizoma*],  *Mentha canadensis* L. [Lamiaceae, *Menthae Haplocalycis Herba*],  *Scutellaria baicalensis* Georgi [Lamiaceae, *Scutellariae Radix*],  *Sophora flavescens* Aiton. [[Fabaceae](https://powo.science.kew.org/taxon/urn:lsid:ipni.org:names:30000147-2), *Sophorae flavescentis Radix*],  *Bassia scoparia* (L.) Beck. [Amaranthaceae, *Kochiae Fructus*],  *Artemisia capillaris* Thunb. [Asteraceae, *Artemisiae Scopariae Herba*],  *Pseudolarix amabilis* (J.Nelson) Rehder. [Pinaceae, *Pseudolaricis Cortex*],  *Gardenia jasminoides* J.Ellis [Rubiaceae, *Gardeniae Fructus*],  *Lonicera confusa* DC. [Caprifoliaceae, *Lonicerae Flos*],  *Phellodendron chinense* C.K.Schneid. [Rutaceae, *Phellodendri Chinensis Cortex*] |
|  |  |
|  |  |
|  |  |
|  |  |
|  |  |
|  |  |
|  |  |
|  |  |
|  |  |
|  |  |
|  |  |
|  |  |
|  |  |
| Kangfu gel (KFG) | *Angelica dahurica* (Hoffm.) Benth. & Hook.f. ex Franch. & Sav. [Apiaceae, *Angelicae Dahuricae Radix*],  *Cnidium monnieri* (L.) Cusson. [Apiaceae, *Cnidii Fructus*],  *Zanthoxylum bungeanum* Maxim. [Rutaceae, *Zanthoxyli Pericarpium*],  *Inula helenium* L. [Asteraceae, *Inulae Radix*],  *Dryobalanops aromatica* C.F.Gaertn. [[Dipterocarpaceae](https://powo.science.kew.org/taxon/urn:lsid:ipni.org:names:77126600-1), *Borneolum*] |
|  |  |
|  |  |
|  |  |
|  |  |
| Kushen gel (KSG) | *Sophora flavescens* Aiton. [[Fabaceae](https://powo.science.kew.org/taxon/urn:lsid:ipni.org:names:30000147-2), Total Alkaloids of *Sophorae flavescentis Radix*] |
| Bai’an lotion (BAL) | *Phellodendron chinense* C.K.Schneid. [Rutaceae, *Phellodendri Chinensis Cortex*],  *Sophora flavescens* Aiton. [[Fabaceae](https://powo.science.kew.org/taxon/urn:lsid:ipni.org:names:30000147-2), *Sophorae flavescentis Radix*],  *Eucalyptus robusta* Sm. [Myrtaceae, *Folium Eucalypti Robustae*],  *Taraxacum mongolicum* Hand. -Mazz. [Asteraceae, *Taraxaci Herba*],  *Bassia scoparia* (L.) Beck. [Amaranthaceae, *Kochiae Fructus*],  *Paeonia lactiflora* Pall. [Paeoniaceae, *Paeoniae Radix Rubra*],  *Cnidium monnieri* (L.) Cusson. [Apiaceae, *Cnidii Fructus*],  *Dryobalanops aromatica* C.F.Gaertn. [[Dipterocarpaceae](https://powo.science.kew.org/taxon/urn:lsid:ipni.org:names:77126600-1), *Borneolum*] |
|  |  |
|  |  |
|  |  |
|  |  |
|  |  |
|  |  |
|  |  |
