# Supplementary material for: Comparative efficacy of topical commercial Chinese polyherbal preparation for vulvovaginal candidiasis: a network meta-analysis
Source: Front Pharmacol. 2025 Feb 3;16:1484325. doi: 10.3389/fphar.2025.1484325 (PMC11830678; doi:10.3389/fphar.2025.1484325)
Supplement: Supplementary file 2 [file Image2.pdf]

Supplementary Figure S2

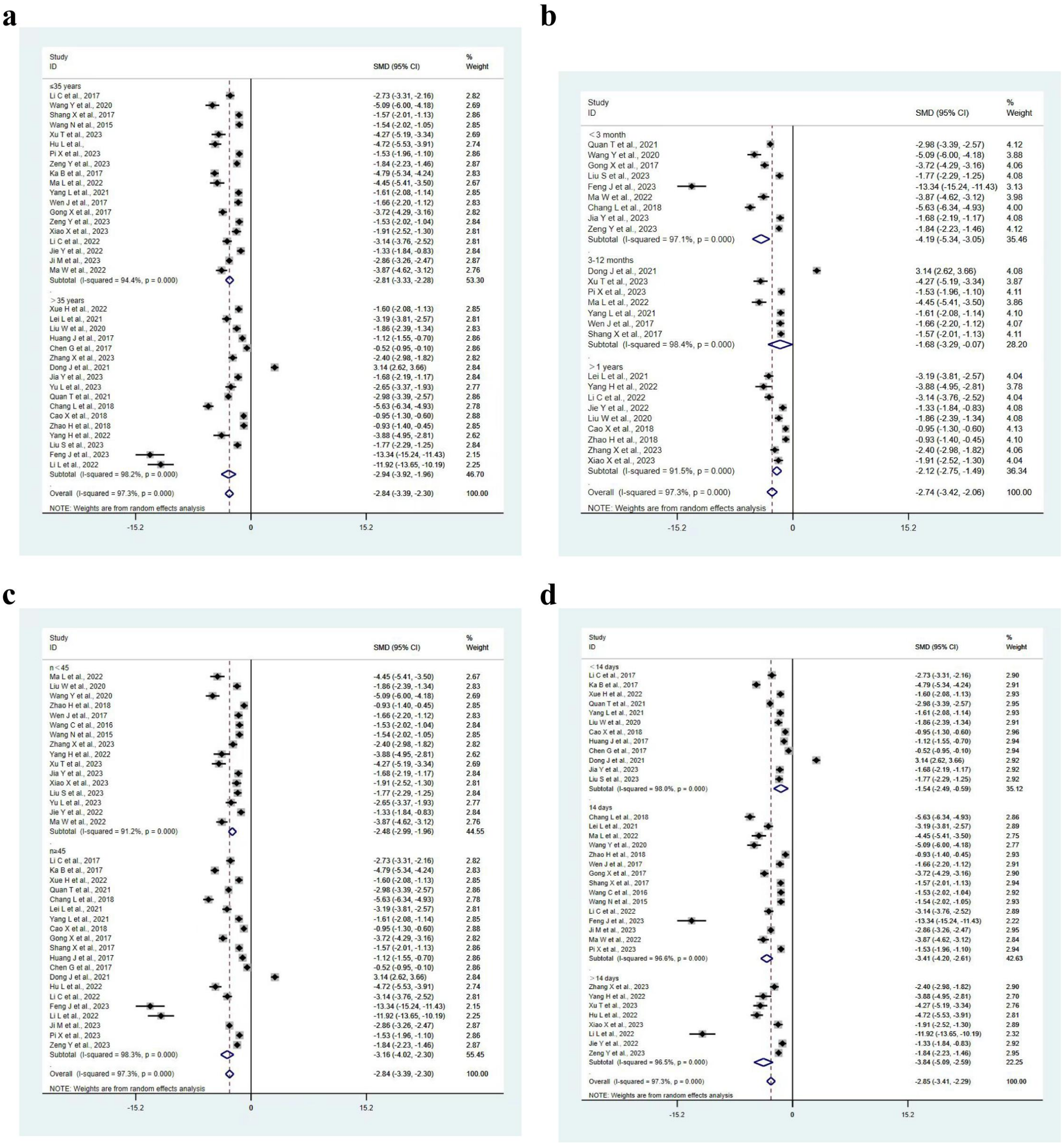

Figure S2: Subgroup analyses of the time to resolution of secretion. (a) age; (b) course of disease; (c) sample size; (d) course of treatment.
