# Supplementary material for: Comparative efficacy of topical commercial Chinese polyherbal preparation for vulvovaginal candidiasis: a network meta-analysis
Source: Front Pharmacol. 2025 Feb 3;16:1484325. doi: 10.3389/fphar.2025.1484325 (PMC11830678; doi:10.3389/fphar.2025.1484325)
Supplement: Supplementary file 3 [file Image3.pdf]

Supplementary Figure S3

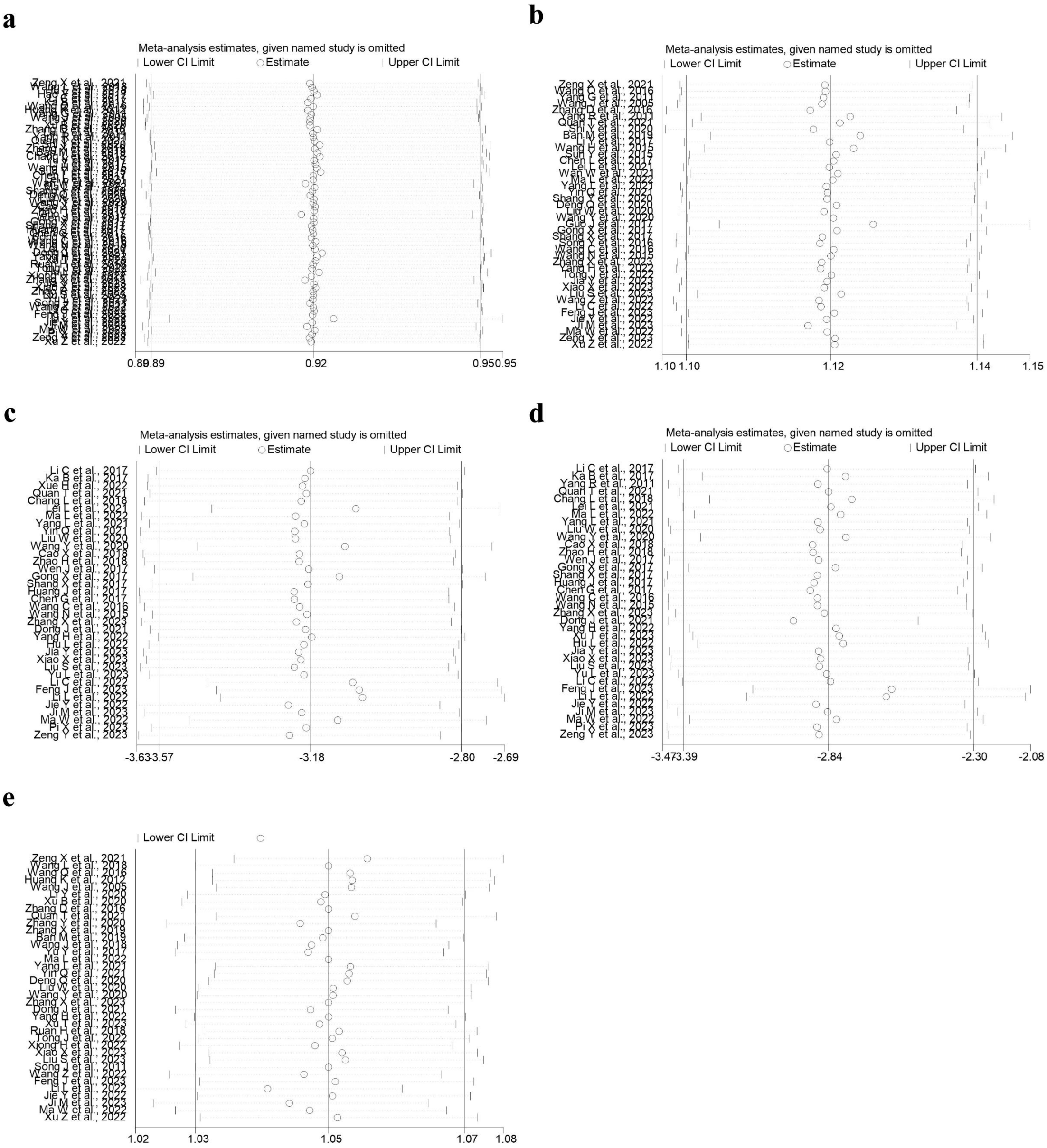

Figure S3: Sensitivity analyses were performed on (a) the negative conversion rate of C. albicans, (b) recurrence rate, (c) time to resolution of pruritus, (d) time to resolution of secretion, and (e) incidence of adverse reactions.
