# Supplementary material for: Comparative efficacy of topical commercial Chinese polyherbal preparation for vulvovaginal candidiasis: a network meta-analysis
Source: Front Pharmacol. 2025 Feb 3;16:1484325. doi: 10.3389/fphar.2025.1484325 (PMC11830678; doi:10.3389/fphar.2025.1484325)
Supplement: Supplementary file 4 [file Table2.docx]

**Table S2. Extract and extraction process description of polyherbal preparations**

| **Drug name** | **Extracts and Extraction Process Description** | **Quality control reported** (Y/N) |
| --- | --- | --- |
| Baofukang suppository (BFKS) | Add an appropriate amount of ethanol to *Curcuma zedoaria* (Christm.) Rosc. [Zingiberaceae, Zedoary Turmeric oil] and *Dryobalanops aromatica* C.F.Gaertn. [[Dipterocarpaceae](https://powo.science.kew.org/taxon/urn:lsid:ipni.org:names:77126600-1), *Borneolum*], and stir until dissolved. Take 1235 g of polyoxyethylene (40) stearate and 200 g of polyethylene glycol 4000, heat until melted, then add 120 g of polyethylene glycol 400 and 17.5g of laurocapram, and mix well. Add the previously prepared medicinal solution, stir well, pour into suppository molds, and cool. Once cooled, remove from the molds to produce 1000 suppositories. | Y.  Z46020058 issued by China Food and Drug Administration |
| Fufukang spray (FFKS) | Soak two botanical drugs in water for 4 ~ 6 h. Then decoct them three times: the first time for 2 h, the second time for 1.5 h, and the third time for 1 h. Filter each time, combine the filtrates, and concentrate to a clear paste with a relative density of 1.13 ~ 1.15 (70 ~ 80 °C). Cool it down, add ethanol to achieve an alcohol content of 90 %, let it stand for 24 h, filter, recover the ethanol from the filtrate until there is no alcohol smell, add Polysorbate 80, then add water to the specified volume, stir well, filter, and package to obtain the final product. | Y.  Z20026246 issued by China Food and Drug Administration |
| Fufang Shajiziyou suppository (FFSJZYS) | First, crush *Cnidium monnieri* (L.) Cusson. [Apiaceae, *Cnidii Fructus*], *Boswellia sacra* Flück. [Burseraceae, *Olibanum*], *Commiphora myrrha* (T.Nees) Engl. [Burseraceae, *Myrrha*], *Sophora flavescens* Aiton. [[Fabaceae](https://powo.science.kew.org/taxon/urn:lsid:ipni.org:names:30000147-2), *Sophorae flavescentis Radix*], and *Smithsonite* [*Calamina*] into a fine powder. Grind *Dryobalanops aromatica* C.F.Gaertn. [[Dipterocarpaceae](https://powo.science.kew.org/taxon/urn:lsid:ipni.org:names:77126600-1), *Borneolum*] into a fine powder and mix it uniformly with the aforementioned powders. Separately, take 1550 g of a glycerin ~ gelatin base and place it in a 75 °C water bath to melt. Add *Hippophae rhamnoides* L. [Elaeagnaceae, *Seabuckthorn Seed Oil*] and 150 mL of water at the same temperature, and quickly emulsify until a viscous gel forms. Incorporate the previously mixed powders, ensuring uniform distribution. Once the bubbles have dissipated, pour the mixture into molds and allow it to cool to form suppositories. | Y.  Z19991076 issued by China Food and Drug Administration |
| Honghe Fujie lotion (HHFJL) | Place the *Crataegus pinnatifida* Bunge. [Rosaceae, *Crataegus Semen*] in a dry distillation kettle and heat it to a temperature range of 100 ~ 120 °C, maintaining this temperature for 30 minutes to ensure the removal of moisture. Continue heating and discard any distillate collected below 150 °C. Collect the dry distillation liquid of *Crataegus Semen* within the temperature range of 150 ~ 270 °C, ensuring the collected amount is between 295 ~ 305 g. Subsequently, add 250 g of Polysorbate to the collected liquid and stir the mixture for 30 minutes to ensure thorough mixing. Add water to the mixture to achieve a total volume of 1000 mL. Stir the final mixture for an additional 30 minutes. Finally, package the mixture into individual containers and seal them to complete the process. | Y.  Z10980131 issued by China Food and Drug Administration |
| Jieeryin lotion (JEYL) | Boil the fourteen botanical drugs in water twice, each time lasting two hours, while concurrently collecting the volatile oil ~ saturated aqueous solution in a separate container. Combine the resulting decoctions, filter the mixture, and concentrate the filtrate to achieve a relative density of 1.022 ~ 1.024. Subsequently, incorporate the previously collected volatile oil ~ saturated aqueous solution, 10 mL of Solubilizer 403, and 2 g of sodium benzoate. Mix thoroughly, adjust the pH to a range of 4.2 ~ 6.0, and add water to bring the total volume to 8000 mL. Ensure the solution is well ~ stirred and then package it into individual containers. | Y.  Z10930008 issued by China Food and Drug Administration |
| kangfu gel (KFG) | Take the four botanical drugs: *Angelica dahurica* (Hoffm.) Benth. & Hook.f. ex Franch. & Sav. [Apiaceae, *Angelicae Dahuricae Radix*], *Cnidium monnieri* (L.) Cusson. [Apiaceae, *Cnidii Fructus*], *Zanthoxylum bungeanum* Maxim. [Rutaceae, *Zanthoxyli Pericarpium*], and *Inula helenium* L. [Asteraceae, *Inulae Radix*]. Add 8 ~ 12 times the amount of water and distill for 2 ~ 4 hours, collecting the aromatic water and the decoction separately.Redistill the aromatic water for 30 ~ 90 minutes to obtain the distillate. Filter the decoction and concentrate the filtrate to a relative density of 1.15 ~ 1.22 at 25 °C. Add ethanol to achieve an alcohol content of 70 %, let it stand, and adjust the pH to 8.0 with a 10 % sodium hydroxide solution. Let it stand overnight, take the supernatant, recover the ethanol, and sterilize to obtain the medicinal liquid. Dissolve disodium edetate in water, then add carbomer, stir well, let it stand for 12 ~ 24 hours, and stir well to obtain the carbomer gel. Take propylene glycol, sequentially add ethanol, azone, ethylparaben, *Borneolum*, the medicinal liquid, and the distillate. Mix well, combine with the carbomer gel, add an appropriate amount of water, stir well to obtain the final product. | Y.  Z20050836 issued by China Food and Drug Administration |
| Kushen gel (KSG) | After crushing the *Sophora flavescens* Aiton. [[Fabaceae](https://powo.science.kew.org/taxon/urn:lsid:ipni.org:names:30000147-2), *Sophorae flavescentis Radix*], the powder was percolated with 0.2 % hydrochloric acid. The percolate’s pH was adjusted to neutral and filtered through a cation exchange resin. The adsorbent on the resin was eluted with ammonia, the eluent was collected, ammonia was recovered, and the eluent was concentrated to dryness to obtain the total alkaloids of sophora flavescens. Dissolve 0.5 ~ 1.5% carbomer (by weight) in purified water and let it swell for 24 h, then stir well. Dissolve 3 ~ 5 % total alkaloids of sophora flavescens (by weight) in ethanol, add propylene glycol to create a solution. Mix the total alkaloids of sophora flavescens solution with the carbomer solution, stir until it forms a gel, adjust the pH to 6 ~ 8, and add purified water to the final volume. | Y.  Z20050058 issued by China Food and Drug Administration |
| Bai’an lotion (BAL) | Utilize steam distillation to extract the volatile oil or prepare the aromatic water from *Bassia scoparia* (L.) Beck. [Amaranthaceae, *Kochiae Fructus*], *Cnidium monnieri* (L.) Cusson. [Apiaceae, *Cnidii Fructus*] and *Eucalyptus robusta* Sm. [Myrtaceae, *Folium Eucalypti Robustae*]. Prepare a decoction by boiling *Phellodendron chinense* C.K.Schneid. [Rutaceae, *Phellodendri Chinensis Cortex*], *Sophorae Flavescentis Radix*, *Taraxacum mongolicum* Hand. -Mazz. [Asteraceae, *Taraxaci Herba*], *Paeonia lactiflora* Pall. [Paeoniaceae, *Paeoniae Radix Rubra*], and the extracted *Kochiae Fructus* and *Cnidii Fructus*. Filter the resulting decoction to obtain the filtrate. Add the previously extracted volatile oil or aromatic water to the filtrate, followed by the addition of propylene glycol, sodium lauryl sulfate, and other solubilizers. Filtrate through an ultrafiltration membrane with an appropriate pore size to obtain the ultrafiltrate. Add *Borneolum* dissolved in an appropriate amount of ethanol to the ultrafiltrate, and adjust the volume with water to 1000 mL to obtain the final product. | Y.  Z20090073 issued by China Food and Drug Administration |
