# Supplementary material for: Comparative efficacy of topical commercial Chinese polyherbal preparation for vulvovaginal candidiasis: a network meta-analysis
Source: Front Pharmacol. 2025 Feb 3;16:1484325. doi: 10.3389/fphar.2025.1484325 (PMC11830678; doi:10.3389/fphar.2025.1484325)
Supplement: Supplementary file 5 [file Table3.docx]

**Table S3. Details about the product information of polyherbal preparations**

| **Drug name** | **Adverse drug reactions** | **Contraindication or Precautions** |
| --- | --- | --- |
| Baofukang suppository (BFKS) | 1. Temporary increase in body temperature or symptoms of chills and shivering may occur after medication, mostly in elderly women or those with low estrogen levels. These symptoms usually subside on their own after discontinuing the medication. (2) Cases of burning sensation, pain, itching, redness, swelling, rash, allergies, and vaginal bleeding at the application site have been reported. These reactions generally gradually alleviate and disappear after discontinuing the medication. | Contraindicated during the first 12 weeks of pregnancy. |
|  |  |  |
| Fufukang spray (FFKS) | Not yet clear. | Not yet clear. |
|  |  |  |
| Fufang Shajiziyou suppository (FFSJZYS) | Occasionally see vulva skin pruritus, accompanied by papules or local redness, generally after stopping the medicine can disappear. | Pregnant women with caution. |
|  |  |  |
|  |  |  |
|  |  |  |
|  |  |  |
| Honghe Fujie lotion (HHFJL) | Not yet clear. | Not yet clear. |
| Jieeryin lotion (JEYL) | Some patients may experience exacerbation of skin redness and prickling pain at the site of skin lesions. | Not to be used by women during menstruation and pregnancy. |
| Kangfu gel (KFG) | Not yet clear. | Not yet clear. |
| Kushen gel (KSG) | Monitoring data indicate that this product may cause adverse reactions such as burning sensation, pain, swelling, itching, discomfort at the local application site, vaginal bleeding, skin itching, rash, and lower abdominal pain. | 1. This product is for vaginal administration only and must not be taken orally. (2) Use with caution in individuals with allergic tendencies. (3) This product is a brown, transparent, jelly-like semi-solid water-soluble gel; do not use if the appearance changes. (4) Use with caution if there are local vaginal lesions. (5) This product contains ethanol; use with caution if allergic to ethanol. (6) Use with caution during pregnancy. (7) Discontinue use during menstruation and resume after menstruation ends. (8) If burning sensation, pain, vaginal bleeding, skin itching, rash, or other discomfort occurs after use, discontinue use immediately and seek medical attention if symptoms are severe. (9) Maintain cleanliness of the vulva during use. (10) Insert the plastic applicator gently into the deep part of the vagina during use. (11) Should not be used concurrently with *Veratri Nigri Radix et Rhizoma* or traditional Chinese medicines containing *Veratri Nigri Radix et Rhizoma*. |
| Bai’an lotion (BAL) | Occasional local discomfort at the application site. During the study period, there was one case of local allergy in the test group, mainly manifested as moderate redness, swelling, heat, and pain of the vulva lasting for 2 days. | 1. This product is for external use only and strictly prohibited for oral use. (2) Avoid contact with eyes and broken skin. (3) Do not use during menstrual periods. (4) Contraindicated in individuals allergic to this product. (5) Not to be used by individuals who have not had sexual intercourse. |
