# Supplementary material for: Comparative efficacy of topical commercial Chinese polyherbal preparation for vulvovaginal candidiasis: a network meta-analysis
Source: Front Pharmacol. 2025 Feb 3;16:1484325. doi: 10.3389/fphar.2025.1484325 (PMC11830678; doi:10.3389/fphar.2025.1484325)
Supplement: Supplementary file 6 [file Table4.docx]

**Table S4. Search strategy used in the Pubmed**

| No. | Time: from the inception until February 29, 2024. |
| --- | --- |
| #1 | Candidiasis, Vulvovaginal [MeSH Terms] |
| #2 | Candidiasis, Vulvovaginal [Title/Abstract] OR Vulvovaginal Candidiasis [Title/Abstract] OR Moniliasis, Vulvovaginal [Title/Abstract] OR Vulvovaginal Moniliasis [Title/Abstract] OR Vaginitis, Monilial [Title/Abstract] OR Monilial Vaginitis [Title/Abstract] OR Candidiasis, Genital [Title/Abstract] OR Genital Candidiasis [Title/Abstract] OR Vaginal Yeast Infections [Title/Abstract] OR Infections, Vaginal Yeast [Title/Abstract] OR Infection, Vaginal Yeast [Title/Abstract] OR Yeast Infections, Vaginal [Title/Abstract] OR Yeast Infection, Vaginal [Title/Abstract] OR Vaginal Yeast Infection [Title/Abstract] OR Genital Vulvovaginal Candidiasis [Title/Abstract] OR Candidiasis, Genital Vulvovaginal [Title/Abstract] OR Vulvovaginal Candidiasis, Genital [Title/Abstract] OR Mycotic vulvovaginitis [Title/Abstract] |
| #3 | #1 OR #2 |
| #4 | Chinese patent medicine [MeSH Terms] OR Chinese patent medicine [Title/Abstract] OR Chinese Patent Drugs [Title/Abstract] OR Chinese Patent Drug [Title/Abstract] OR Fufang Shajiziyou [Title/Abstract] OR Kushen [Title/Abstract] OR Kangfu [Title/Abstract] OR Baofukang [Title/Abstract] OR Fufukang [Title/Abstract] OR Honghe Fujie [Title/Abstract] OR Bai’an [Title/Abstract] OR Jieeryin [Title/Abstract] |
| #5 | Randomized Controlled Trial [Publication Type] OR Controlled Clinical Trial [Publication Type] OR random [All Fields] OR randomised [All Fields] OR randomized [All Fields] |
| #6 | #3 AND #4 AND #5 |

**Table S5. Search strategy used in the Web of science**

| No. | Time: from the inception until February 29, 2024. |
| --- | --- |
| #1 | Candidiasis, Vulvovaginal (Topic) or Vulvovaginal Candidiasis (Topic) or Moniliasis, Vulvovaginal (Topic) or Vulvovaginal Moniliasis (Topic) or Vaginitis, Monilial (Topic) or Monilial Vaginitis (Topic) or Candidiasis, Genital (Topic) or Genital Candidiasis (Topic) or Vaginal Yeast Infections (Topic) or Infections, Vaginal Yeast (Topic) or Infection, Vaginal Yeast (Topic) or Yeast Infections, Vaginal (Topic) or Yeast Infection, Vaginal (Topic) or Vaginal Yeast Infection (Topic) or Genital Vulvovaginal Candidiasis (Topic) or Candidiasis, Genital Vulvovaginal (Topic) or Vulvovaginal Candidiasis, Genital (Topic) or Mycotic vulvovaginitis (Topic) |
| #2 | Chinese patent medicine (Topic) or Chinese Patent Drugs (Topic) or Chinese Patent Drug (Topic) or Fufang Shajiziyou (Topic) or Kushen (Topic) or Baofukang (Topic) or Fufukang (Topic) or Honghe Fujie (Topic) or Bai’an (Topic) or Jieeryin (Topic) |
| #3 | Randomized Controlled Trial (Topic) or Controlled Clinical Trial (Topic) or randomised (Topic) or randomized (Topic) or random (Topic) |
| #4 | #1 AND #2 AND #3 |

**Table S6. Search strategy used in the embase**

| No. | Time: from the inception until February 29, 2024. |
| --- | --- |
| #1 | vulvovaginal candidiasis/exp |
| #2 | 'vulvovaginal candidiasis': ti,ab,kw OR 'candida vulvovaginitis': ti,ab,kw OR 'candidal vulvovaginitis': ti,ab,kw OR 'candidiasis, vulvovaginal': ti,ab,kw OR 'monilial vulvovaginitis': ti,ab,kw OR 'vulvo-vaginal candidiasis': ti,ab,kw OR 'vulvo-vaginal candidosis': ti,ab,kw OR 'vulvovaginal candidosis': ti,ab,kw OR 'vulvovaginal moniliasis': ti,ab,kw OR 'vulvovaginitis caused by Candida': ti,ab,kw OR 'vulvovaginitis due to Candida': ti,ab,kw |
| #3 | #1 AND #2 |
| #4 | chinese patent medicine/exp |
| #5 | 'chinese patent medicine': ti,ab,kw OR Chinese Patent Drug: ti,ab,kw OR Fufang Shajiziyou: ti,ab,kw OR Kushen: ti,ab,kw OR Baofukang: ti,ab,kw OR Fufukang: ti,ab,kw OR Honghe Fujie: ti,ab,kw OR Baian:ti,ab,kw OR Jieeryin: ti,ab,kw |
| #6 | #4 AND #5 |
| #7 | randomized controlled trial/exp |
| #8 | 'randomized controlled trial': ti,ab,kw OR 'controlled trial, randomized': ti,ab,kw OR 'randomised controlled study': ti,ab,kw OR 'randomised controlled trial': ti,ab,kw OR 'randomized controlled study': ti,ab,kw OR 'trial, randomized controlled': ti,ab,kw |
| #9 | #7 AND #8 |
| #10 | #3 AND #6 AND #9 |

**Table S7. Search strategy used in the Cochrane Library**

| No. | Time: from the inception until February, 2024. |
| --- | --- |
| #1 | MeSH descriptor: [Candidiasis, Vulvovaginal] explode all trees |
| #2 | (Candidiasis, Vulvovaginal): ti,ab,kw OR (Candidiasis, Genital Vulvovaginal): ti,ab,kw OR (Vulvovaginal Candidiasis, Genital): ti,ab,kw OR (Genital Vulvovaginal Candidiasis): ti,ab,kw OR (Vulvovaginal Moniliasis): ti,ab,kw OR (Moniliasis, Vulvovaginal): ti,ab,kw OR (Vulvovaginal Candidiasis): ti,ab,kw OR (Vaginal Yeast Infections): ti,ab,kw OR (Infection, Vaginal Yeast): ti,ab,kw OR (Yeast Infections, Vaginal): ti,ab,kw OR (Infections, Vaginal Yeast): ti,ab,kw OR (Yeast Infection, Vaginal): ti,ab,kw OR (Vaginal Yeast Infection): ti,ab,kw OR (Monilial Vaginitis): ti,ab,kw OR (Vaginitis, Monilial): ti,ab,kw OR (Genital Candidiasis): ti,ab,kw OR (Candidiasis, Genital): ti,ab,kw |
| #3 | #1 OR #2 |
| #4 | (Chinese patent medicine): ti,ab,kw OR (Chinese Patent Drug): ti,ab,kw OR (Fufang Shajiziyou): ti,ab,kw OR (Kushen): ti,ab,kw OR (Baofukang): ti,ab,kw OR (Fufukang): ti,ab,kw OR (Honghe Fujie): ti,ab,kw OR (Baian): ti,ab,kw OR (Jieeryin): ti,ab,kw |
| #5 | MeSH descriptor: [Randomized Controlled Trial] explode all trees |
| #6 | (Randomized Controlled Trial):ti,ab,kw OR (Controlled Clinical Trial):ti,ab,kw OR (randomised):ti,ab,kw OR (random):ti,ab,kw |
| #7 | #5 OR #6 |
| #8 | #3 AND #4 AND #7 |

**Table S8. Search strategy used in** **China National Knowledge Infrastructure (CNKI)**

| No. | Time: from the inception until 29th February 2024. |
| --- | --- |
| #1 | SU %= '念珠菌病, 外阴阴道' OR TKA = '外阴阴道假丝酵母菌病' OR TKA = '外阴阴道念珠菌性疾病' OR TKA = '念珠菌病, 外阴阴道' OR TKA = '阴道炎, 念珠菌性' OR TKA = '念珠菌性阴道炎' OR TKA = '外阴阴道念珠菌病' OR TKA = '霉菌性阴道炎' |
| #2 | SU %= '中成药' OR TKA = '中成药' OR TKA = '外用药' OR TKA = '复方沙棘籽油栓' OR TKA = '苦参凝胶' OR TKA = '康妇凝胶' OR TKA = '保妇康栓' OR TKA = '妇肤康喷雾剂' OR TKA = '红核妇洁洗液' OR TKA = '百安洗液' OR TKA = '洁尔阴洗液' |
| #3 | SU %= '随机' OR TKA = '随机' OR TKA = '随机分组' OR TKA = '临床观察' OR TKA = '观察' OR TKA = '疗效' |
| #4 | #1 AND #2 AND #3 |

**Table S9. Search strategy used in Wanfang Database**

| No. | Time: from the inception until February 2024. |
| --- | --- |
| #1 | 主题: (念珠菌病, 外阴阴道 or 外阴阴道假丝酵母菌病 or 外阴阴道念珠菌性疾病 or 阴道炎, 念珠菌性 or 念珠菌性阴道炎 or 外阴阴道念珠菌病 or 霉菌性阴道炎) |
| #2 | 主题: (中成药 or 外用药 or 复方沙棘籽油栓 or 苦参凝胶 or 康妇凝胶 or 保妇康栓 or 妇肤康喷雾剂 or 红核妇洁洗液 or 百安洗液 or 洁尔阴洗液) |
| #3 | 主题: (随机对照 or 随机 or RCT or 观察 or 临床观察 or 疗效) |
| #4 | #1 AND #2 AND #3 |

**Table S10. Search strategy used in China Biology Medicine disc (CBM)**

| No. | Time: from the inception until February 2024. |
| --- | --- |
| #1 | ("外阴阴道假丝酵母菌病"[常用字段:智能] OR "外阴阴道念珠菌性疾病"[常用字段:智能] OR "念珠菌性阴道炎"[常用字段:智能] OR "阴道炎&念珠菌性"[常用字段:智能] OR "念珠菌病&外阴阴道"[常用字段:智能] OR "外阴阴道念珠菌病"[常用字段:智能] OR "霉菌性阴道炎"[常用字段:智能]) AND -2024[日期] |
| #2 | ("中成药"[常用字段:智能] OR "复方沙棘籽油栓"[常用字段:智能] OR "苦参凝胶"[常用字段:智能] OR "康妇凝胶"[常用字段:智能] OR "保妇康栓"[常用字段:智能] OR "妇肤康喷雾剂"[常用字段:智能] OR "红核妇洁洗液"[常用字段:智能] OR "百安洗液"[常用字段:智能] OR "洁尔阴洗液"[常用字段:智能]) AND -2024[日期] |
| #3 | ("随机对照"[常用字段:智能] OR "随机"[常用字段:智能] OR "RCT"[常用字段:智能] OR "观察"[常用字段:智能] OR "临床观察"[常用字段:智能] OR "疗效"[常用字段:智能]) AND -2024[日期] |
| #4 | #1 AND #2 AND #3 |

**Table S11. Search strategy used in the Chinese Scientific Journal Database (VIP)**

| No. | Time: from the inception until February 2024. |
| --- | --- |
| #1 | M= (念珠菌病, 外阴阴道 or 外阴阴道假丝酵母菌病 or 外阴阴道念珠菌性疾病 or 阴道炎, 念珠菌性 or 念珠菌性阴道炎 or 外阴阴道念珠菌病 or 霉菌性阴道炎) |
| #2 | M= (中成药 or 外用药 or 复方沙棘籽油栓 or 苦参凝胶 or 康妇凝胶 or 保妇康栓 or 妇肤康喷雾剂 or 红核妇洁洗液 or 百安洗液 or 洁尔阴洗液) |
| #3 | M= (随机对照 or 随机 or RCT or 观察 or 临床观察 or 疗效) |
| #4 | #1 AND #2 AND #3 |
